# Supplementary material for: High prevalence of hepatitis B virus infection among pregnant women attending antenatal care: a cross-sectional study in two hospitals in northern Uganda
Source: BMJ Open. 2014 Nov 11;4(11):e005889. doi: 10.1136/bmjopen-2014-005889 (PMC4244481; doi:10.1136/bmjopen-2014-005889)
Supplement: Author's manuscript [file bmjopen-2014-005889.draft_revisions.pdf]

BMJ Open

High prevalence of Hepatitis B virus infection among pregnant women attending antenatal care: a cross-sectional study in two referral hospitals in northern Uganda

Journal:	*BMJ Open*
Manuscript ID:	bmjopen-2014-005889
Article Type:	Research
Date Submitted by the Author:	11-Jun-2014
Complete List of Authors:	Bayo, Pontius; St. Mary's Hospital Lacor, Obstetrics and Gynecology<br>Ochola, Emmanuel; Gulu University Faculty of Medicine, Public Health<br>Oleo, Carolyne; Makerere University College of Health Sciences, School of Medicine, Obstetrics & Gynecology<br>Mwaka, Amos; Mulago Hospital/Makerere University, Department of Internal Medicine
**Primary Subject Heading**:	Infectious diseases
Secondary Subject Heading:	Obstetrics and gynaecology
Keywords:	Infection control < INFECTIOUS DISEASES, Prenatal diagnosis < OBSTETRICS, PREVENTIVE MEDICINE, Hepatobiliary tumours < ONCOLOGY

SCHOLARONE™
Manuscripts

For peer review only

High prevalence of Hepatitis B virus infection among pregnant women attending antenatal care: a cross-sectional study in two hospitals in northern Uganda

Corresponding author: Pontius Bayo, St. Mary's Hospital Lacor, P.O. Box 180, Gulu, Uganda.

Email: pontiusby@gmail.com Tel. +256 782 1424 35

Co- authors:

Emmanuel Ochola[1,2], Caroline Oleo[2,3], Amos Deogratius Mwaka[3]

1. St. Mary's Hospital Lacor, P.O. Box 180, Gulu, Uganda. 2. Gulu University Medical School, P.O. Box 166, Gulu, Uganda. 3. Makerere University College of Health Sciences, School of Medicine, P. O. Box 7072, Kampala, Uganda.

For peer review only

Key words: Hepatitis B virus, Prevalence, Pregnant women, Northern Uganda

Word count: 3,452.

ABSTRACT

Objective: To determine prevalence of hepatitis B viral (HBV) infection and Hepatitis B e antigen (HBeAg) positivity among pregnant women attending antenatal clinics in two referral hospitals in northern Uganda.

Design: Cross-sectional observational study.

Participants: Randomly selected 402 pregnant women attending routine antenatal care in two referral hospitals, however, five withdrew consent for personal reasons. Data was analyzed for 397 participants.

Setting: Tertiary hospitals in a post-conflict region in low-income country.

Results: Of 397 pregnant women, 47 (11.8%) tested positive for hepatitis B surface antigen (HBsAg); of these, 7 (14.9 %) were HBeAg positive. The highest HBsAg positivity rate was seen in women aged 20 years or less (20%) compared to those above 20 years old (8.7%), aOR = 2.54 (955 CI: 1.31 - 4.90) and the difference was statistically significant(p = 0.006). However, there was no statistically significant difference between women with positive HBsAg versus negative tests with respect to liver enzymes levels, hemoglobin level, absolute neutrophil counts and mean white blood cells (WBC) counts. HIV positivity, scarification, and number of sexual partners were not predictive of HBV positivity.

Conclusion: One in eight pregnant women attending care in the two study hospitals is Hepatitis B infected. A significant number of these mothers are HBe Ag positive and may be at an increased risk of transmitting hepatitis B infection to their unborn babies. We suggest that all pregnant women attending ante natal care be tested for hepatitis B viral infection; exposed babies need to receive HBV vaccines at birth. Catch up vaccination to reduce burden of HBV infection

For peer review only

For peer review only - http://bmjopen.bmj.com/site/about/guidelines.xhtml

in the community is recommended for adolescence and young adults who missed HBV immunizations in childhood.

Strengths and limitations of this study

- This study has evaluated the prevalence of a sexually transmitted viral infection, a risk factor for hepatocellular carcinoma in a population exposed to no condom sexual intercourse in a post-conflict region with high rates of HIV infection, another surrogate marker for sexually transmitted infections.
- This study has also investigated the prevalence of hepatitis B e antigen test, a surrogate measure of the risk of vertical transmission of hepatitis B infection.
- The study provides useful information for policy formulation about routine testing of pregnant women and immunization of exposed babies at birth rather than the current practice of using combined vaccine at six weeks.
- The study had some limitations; it was hospital-based and included a selected population with respect to sexually transmitted infection. In addition we could not perform tests for Hepatitis B core antibodies (anti-HBc) and HBV DNA because of logistical reasons.

For peer review only - http://bmjopen.bmj.com/site/about/guidelines.xhtml

INTRODUCTION

Four hundred million people in the world are living with chronic hepatitis B virus (HBV) infection.[1] The majority of these individuals acquired the infection during the peri-natal period.[2] The risk of becoming a chronic Hepatitis B infection carrier is 95% for infections acquired during the perinatal period[3] compared to only 5% for those acquired during adulthood.[4] Up to 50% of HBV carriers die of complications including liver cirrhosis and hepatocellular carcinoma.[5]

Pregnant mothers who test positive for both Hepatitis B surface antigen (HBsAg) and Hepatitis B e antigen (HBeAg) have 70 - 90% risk of transmitting infection to their new borne and about 10 - 40% if they test positive for only HBsAg.[5, 6] Therefore, pregnant women should be routinely screened for HBsAg and Hepatitis B vaccine administered at birth to the infants whose mothers test positive.[7, 8] However, this is not the practice in Uganda.

The Uganda National Expanded Program on Immunizations (UNEPI) scaled-up childhood immunizations in 2002,[9] incorporating the hepatitis B vaccine into a combination vaccine whose first dose is administered at six weeks of age. The six weeks window both limits the efficacy of the vaccine in the prevention of vertical transmission and also allows for the potential transmission of HBV through close contacts.[7] The most effective method of preventing HBV infection is through immunization which offers over 95% protection against the development of chronic infection.[10] Such immunization should be done at birth for exposed infants. There is no evidence of protection against perinatal transmission if the first dose of vaccine is given more than seven days after birth.[11]

In Nigeria, the prevalence of HBV infection among pregnant women was 11% with HbeAg positivity of 33%.[12] In northern Uganda, there is limited knowledge on the prevalence of

For peer review only - http://bmjopen.bmj.com/site/about/guidelines.xhtml

hepatitis B infection among pregnant women. The civil war in this region between the government of Uganda and the Lord's resistance army from the late 1980's up to 2006 led to the displacement of as many as 1.7 million people from their homes into internally displaced persons (IDP) camps.[13] These camps were crowded, traditional and social structures were disrupted and sexually transmitted infections such as HBV seemed to increase. The Uganda HIV sero-behavioral survey of 2004/2005 estimated the prevalence of hepatitis B in northern Uganda to be between 18.4% and 24.3%, much higher than the national average of 10%,[14] while a recent community based study in Gulu municipality estimated the prevalence of HBV in the general population at 17.6%.[15]

In this study we report the prevalence of HBV infection among pregnant women attending antenatal care (ANC) at St. Mary's Hospital Lacor (Lacor) and Gulu Regional referral Hospital using HBsAg test. We also report HBeAg positivity, a surrogate measure of infectivity among those women who tested positive for HBsAg and describe the factors associated with HBV infection among these women.

For peer review only

METHODS

Study design and setting

This was a cross-sectional study at Lacor and Gulu regional referral Hospitals. The two hospitals are both in Gulu district in northern Uganda. Lacor hospital is 6 kilometers west of Gulu town; it is a 482 bed capacity teaching Hospital[16] and a sentinel site for infectious disease surveillance in northern Uganda, and has a laboratory with capacity for the separation and storage of frozen plasma. Lacor Hospital antenatal clinic (ANC) is visited by 50 - 80 pregnant women per day, Monday through Friday. Gulu regional referral Hospital on the other hand, is a 250-beds government owned referral facility located in the center of Gulu town;[16] the antenatal clinic in Gulu hospital is visited by about 40 - 60 pregnant women every working day.

Study population

We included pregnant women attending ante natal care at the two study hospitals during September, 2012 through January, 2013. The two hospitals receive majority of pregnant women from Gulu district; however some women attend ante natal care in other private facilities in the town and health centers.

Sample size and sampling method

We used the Kish Leslie formula (1965) and a prevalence of HBsAg in North-central Uganda of 21% for sample size determination. We allowed for an additional 10% prevalence to account for variations due to the fact that pregnant women are a selected population with higher risk of sexually transmitted infections compared to the general population.[14] A further 10% was added to cater for possible incomplete responses; hence 402 participants were recruited.

For peer review only

Sampling procedures

Women were sampled on two working days a week in the two study hospitals; Lacor on Wednesdays and Fridays while in Gulu, sampling was done on Mondays and Thursdays. All pregnant women attending ante natal care on the study days were verbally informed of the study immediately after the routine ante natal care health education. They were informed that not all women will be selected because particular criteria of selection, not disclosed to them will be used. Systematic random sampling, selecting every 5th woman on the ante natal care waiting line was done. We excluded women who had emergency conditions requiring urgent intervention.

Data collection procedures

At each study site, two midwives were trained for two days on study procedures, facts on HBV infections and transmissions, counseling, safety issues, sample collection and transportation as well as site testing for HBsAg.

Upon obtaining written informed consents, a questionnaire was administered to every selected woman to obtain socio-demographic information including maternal age, gestation age, gravidity, occupation, marital status and highest level of education. Other information on risk factors for transmission of HBV including history of previous blood transfusions, and history of scarification were also obtained. The women were then helped to immediately receive care from the clinic staffs.

Participants were informed that those who test positive for HBsAg would be informed and called back to receive results of another test (HBeAg) to be done on their stored blood samples. They were also counseled about the hepatitis B vaccine that the study would provide to their infants at

For peer review only - http://bmjopen.bmj.com/site/about/guidelines.xhtml

birth. Plasma-derived hepatitis B vaccine was administered to infants born to HBsAg positive mothers within 12 hours of birth. Each vaccine dose (0.5 ml) contained 10 pg of purified HBsAg.

Laboratory procedures

Trained research assistants provided pre-test counseling on hepatitis B viral and HIV infections. Five milliliters (5 mls) of blood was then drawn by venipuncture from the cubital fossa under aseptic techniques. The blood samples were immediately put into portable cold boxes with ice packs. The research assistants immediately transported samples to the laboratories at study sites to test for HBsAg test (i.e. at Lacor and Gulu Hospital Laboratories). In the meantime, the women were helped to obtained ante natal care. Results were collected back by the research assistants who provided post-test counseling and released results to the participants on the same visit day. Blood samples positive for HBsAg from Gulu hospital were transported on same day to Lacor hospital laboratory and were frozen at -80°C and later transferred to MBN Clinical laboratories in the capital Kampala, for HBeAg testing.

Testing for HBsAg was done using the Infectious Diseases Enzyme Linked Immunosorbent Assay (ELISA) kits which has sensitivity of 100% and specificity of 99.7%. Testing for HbeAg was done using the Infectious Diseases ELISA - peroxidase conjugated kits, which have 100% sensitivity and 99.9% specificity, and inbuilt quality controls.

Samples from all the participants were tested for HIV, complete blood counts (CBC), liver alanine aminotransferase (ALT), aspartate aminotransferase (AST), alkaline phosphatase (ALP), using a SMAC auto-analyzer (Semi Micro Analyzer Computer, Technicon, USA). Complete blood count was done with an automated analyzer, Humacount 60^{TS}. HIV tests were performed using rapid assay for HIV antibody testing.

For peer review only - http://bmjopen.bmj.com/site/about/guidelines.xhtml

Data analysis

Descriptive data included proportions, means and medians. Statistical analysis was done using STATA software version 12.0. Association between demographic variables and laboratory parameters were assessed using chi-square; logistic regressions were carried to determine magnitudes of associations; differences in means were assessed using the student t-tests. A p-value of ≤ 0.05 was considered statistically significant in all statistical tests.

Ethical considerations

Each prospective participant received explanation about the study in their language of choice, mostly Acholi, the major Ugandan language spoken in the study region. They were provided with and given 20 minutes to study the IRC stamped consent forms in the local language; and thereafter, requested for their informed consents to participate in the study. Questionnaires were administered only after signed or thumb printed consents. All participants did not pay for tests done, and test results were provided to the women. All infants born to mothers positive for HBsAg received hepatitis B vaccines at the costs of the study team. Institutional ethical approval was sought from Lacor hospital Ethics Review Committees (IRC), and the Uganda National Council of Science and Technology (UNCST).

For peer review only

RESULTS

Study participants

We approached 402 participants (200 from Lacor and 202 from Gulu Hospital). Five mothers withdrew consent; we therefore, included 397 participants in the analysis.

The median age of the participants was 24 years (range: 13-43 years). Regarding ethnicity, 89% of the participants were of Acholi tribe. Up to 96.2% of the women were either married or cohabiting; 71.8% of the married women were in monogamous relationship (Table 1).

For peer review only

Table 1: Socio-demographic characteristics of 397 antenatal Hepatitis B study participants

Variable	Frequency, N=397	Percent (%)
**Age**		
≤ 20 years	110	27.7
>20 years	287	72.3
**Education**		
None	30	7.6
Primary	191	48.0
Secondary	140	35.3
Tertiary	41	9.1
**Tribe**		
Acholi	356	89.7
Lango	17	4.3
Others*	24	6.0
**Occupation**		
Peasant	220	55.4
Professional	44	11.1
Other	133	33.5
**Marital status**		
Single	15	3.8
Married	160	40.3
Cohabiting	222	55.9
**Type of marriage**		
Monogamous	285	71.8
Monogamous	112	28.2
**Parity**		
0	93	23.4
1-4	250	63.0
5+	54	13.6
**HIVstatus**		
Negative	360	90.7
Positive	37	9.3
**Scarification**		
Not Done	43	10.8
Done	354	89.2

*Other tribes include Madi, Baganda, Jalwo, Karimojong, Banyoro

For peer review only

Prevalence hepatitis B, HIV and HBeAg positivity

The overall prevalence of Hepatitis B surface antigen (HBsAg) positivity was 11.8%; the prevalence was 12.7% and 10.9% in Lacor and Gulu Hospitals respectively (Table 2). Hepatitis B e Antigen was positive in 7 of the 47 HBsAg positive women (14.9%).

Hepatitis B surface antigen positive mothers were significantly younger than the negative mothers ($p = 0.002$) (Table 2).

The prevalence of HIV infection among the participants was 9.3% but there was no statistically significant difference in Hepatitis B prevalence by HIV status.

For peer review only

For peer review only - http://bmjopen.bmj.com/site/about/guidelines.xhtml

Table 2: Association between socio-demographic and clinical characteristics of HBsAg positive mothers

Variable (n)	Hepatitis B infection (HBsAg positive)			
	N positive	%	Crude OR (CI)	P value
**Overall (397)**	47*	11.8	--	--
**Age**				
≤ 20 years (110)	22	20	2.62 (1.41-4.89)	0.002**
>20 years (287)	25	8.7	ref	
**Education**				
None (30)	6	20	ref	
Primary (191)	17	8.9	0.39 (0.14-1.09)	0.070
Secondary(140)	20	14.3	0.67 (0.24-1.83)	0.432
Tertiary (36)	4	11.8	0.50 (0.13-1.97)	0.322
**Marital status**				
Not married (237)	30	12.7	ref	
Married	17	10.6	0.82 (0.44-1.54)	0.539
**Parity**				
0 (93)	13	14.0	ref	
1-4 (250)	29	11.6	0.81 (0.40-1.63)	0.551
5+ (54)	5	9.3	0.63 (0.21-1.87)	0.403
**HIVstatus**				
Negative (360)	43	11.94	ref	
Positive (37)	4	10.81	0.89 (0.30-2.65)	0.839
**Scarification**				
Not Done (43)	5	10.6	ref	
Done (354)	42	11.2	1.02 (0.38-2.74)	0.934
**History of Blood transfusion**				
No (383)	44	11.5	ref	
Yes (14)	3	21.43	2.10(0.56-7.82	0.268
**No. of sexual partners last 2yrs**				
1 (377)	43	11.4	ref	
2 (20)	4	20.0	1.93 (0.62-6.08)	0.254

*Of the 47 with HBsAg positive result, 7 (14.9%) were found Hepatitis B e Antigen positive

**At Multivariate level, only age remained significant predictor of HBsAg positivity, at aOR=2.54 (1.31-4.90); p value 0.006.

For peer review only

Hepatitis B risk factors

Common risk factors like history of scarification, number of sexual partners, history of blood transfusion or polygamy had no statistically significant relationship with HBsAg positivity (table 2). The liver function tests and complete blood cell counts were similar in both HBs Ag positive and negative women. Majority of women had hemoglobin concentrations (Hb) and platelets counts within normal ranges; these counts were not predictive of HBsAg positivity (table 3).

Table 3: Laboratory test results for HBsAg positive and negative mothers

	HBsAg positive	HBsAg negative	p-value
Mean WBC (SD)	6535.96 (2004.26)	6345.46 (1824.65)	0.506
Mean Lymphocytes (SD)	1901.28 (725.07)	1665.37 (725.07)	0.062
Neutrophils (SD)	4247.45 (1628.74)	4104.48 (1492.48)	0.940
Haemoglobin (SD)	12.07 (0.93)	14.15 (13.36)	0.485
ALP	282.74 (125.28)	279.58 (137.04)	0.880
AST	22.91 (8.49)	22.49 (14.63)	0.846
ALT	21.68 (11.75)	21.44 (18.40)	0.933

For peer review only

For peer review only - http://bmjopen.bmj.com/site/about/guidelines.xhtml

On multivariable analysis, women 20 years of age or younger were 2.5 folds more likely to test positive than those aged over 20 years; aOR 2.52, CI (1.31-4.90); p value 0.006) (Table 2 footnote).

For peer review only

For peer review only - http://bmjopen.bmj.com/site/about/guidelines.xhtml

DISCUSSION

This study highlights the high prevalence of Hepatitis B virus infection (11.8%) among pregnant women attending ante natal care in two hospitals in the post-conflict region of northern Uganda. Although the prevalence of both HBV and HIV infections in this region exceeds those in most other regions of Uganda that have not experienced prolonged civil conflict and internment in camps, no causal relationship between HBV infection and civil conflict can be inferred from this findings from a cross-sectional study. We also found that about 15% of the HBsAg positive mothers were also HBeAg positive. The prevalence of HBV infection was higher among women aged 20 years or younger (20%) than among the older women (8.7%). HIV infection among the study population was also high (9.3%). However there was no significant association between HIV infection and HBV infection among the pregnant women included in this study. Thus about one in every eight pregnant women in Gulu is Hepatitis B infected.

The prevalence of HBV infection among pregnant women in this study is consistent with findings from a study in Nigeria which found a prevalence of 11%, and a higher HBeAg positivity of 33%.[12] The majority of people who get HBV infection after neonatal period tend to clear the virus over time; it is therefore, conceivable for pregnant women to be at HBsAg positivity similar to the general population from which they come. Our findings add evidence to the fact that HBV infection rate might be high in the general population of northern Uganda. The finding in this study that 3 in 20 pregnant women with positive HBsAg are also HBeAg positive means that the unborn babies of these pregnant women are at high risk of infection with HBV. The infants of these mothers will need immediate vaccination with HBV vaccine upon delivery. This is however not the practice in Uganda and that means the risk of infection is not adequately

For peer review only - http://bmjopen.bmj.com/site/about/guidelines.xhtml

minimized in these infants. Children who contract HBV infections from their mothers are more likely to develop chronic HBV infection and progress to liver complications associated with active HBV infection including cirrhosis and hepatocellular carcinoma.

To demonstrate a need for a specific affirmative program to reduce the incidence of complications from chronic HBV infections in this community, we discuss our findings in context of HBV infections in Uganda as a whole. Review of the sentinel surveillance data shows that the HBV infections prevalence in this study is higher than HBV infection prevalence among the high risk HIV positive pregnant women (4.9%) in central Uganda[17] and among HIV infected adult population (5%) in Rakai, south western Uganda[18]. The prevalence of HBV infection of 18 - 24% in the general population in northern Uganda is in fact higher than in most parts of Uganda,[14, 19] and so the findings in this study for the pregnant population just mirrors the background population prevalence in northern Uganda. It is important that special attention is paid to the high incidence of infection in this region in order to reduce the cost of care of chronic liver diseases including hepatocellular carcinoma in the future. We suggest that government of Uganda and development partners involved in healthcare planning and provisions in Uganda urgently introduce routine screening for HBV infection during pregnancy and vaccination at birth for the exposed infants, in the northern region in order to reduce incidences of peri-natal infections with HBV.

In this study, the prevalence of HBV infection was higher among the younger compared to the older women. This is in variance to findings from a study in Mauritania where there was no significant difference in the mean age of pregnant women who were HBsAg positive compared to those who were negative.[20] Our finding is however, similar to results from the Uganda national sero-behavioral survey in 2005 which showed a prevalence of 8.8% in the age group 15-

For peer review only - http://bmjopen.bmj.com/site/about/guidelines.xhtml

19 years of age and increments with age[19] and in Rakai where positive HBsAg tests reached the highest at 8% among the age group 20-29 years of age.[18] The high prevalence of HBV infection among the younger age group in this study and in the general Ugandan population may be related to the relatively high vulnerability of the younger women to sexually transmitted infections.[21] A study by Råssjö et al, showed that females were more likely to be infected by sexually transmitted infections (STIs) despite risky behavior being more common among males[22] and that biological and social factors especially unemployment and little formal education contribute significantly to higher prevalence of STIs, including Hepatitis B, among adolescent girls. However, in this study there were no significant differences in employment status, education levels, marital status and number of sexual partners in the previous two years among HBsAg positive participants and those who were negative.

The risk of vertical transmission of HBV infection to the unborn child may be related to the effect of HBV infection on the mother; how she responds to the infection, the timing of the infection with respect to the current pregnancy and the immune status of the mother as well as the levels of HBV DNA.[23] The probability of vertical infection is however, much increased when the mother is also HBeAg.[3] In one study Vertical transmission was seen in 65% of babies born to mothers who were positive for HBeAg and in 9.1% for babies born to mothers who were negative for HBeAg.[24] In Senegal, out of 21 infants born to HBsAg positive mothers 11 were HBsAg positive at birth, and at 6 -7 months, five of these were still strongly HBsAg positive and developed antibodies to HBsAg, HBcAg or HBeAg.[25] At present, pregnant women in Uganda are not routinely screened for HBsAg, and the exposed newborns are not immunized at birth against HBV infection. This high prevalence rate of HBsAg positivity among asymptomatic pregnant women in our study shows that there are many infants born who are at high risk of

For peer review only - http://bmjopen.bmj.com/site/about/guidelines.xhtml

becoming chronic Hepatitis B carriers and dying of chronic liver disease at young adult age in future.

This study is not without limitation. We did not test for anti-HBc and HBV DNA; and so there could have been HBsAg negative individuals with isolated anti-HBc and occult HBV infection. However, a recent study among HIV infected pregnant women showed that pregnant women with isolated anti-HBc and occult HBV infection have very low HBV DNA levels and are thus at very low risk to transmit HBV to their infants.[26] We also did not perform high resolution abdominal ultrasound scans nor did we carry out serial liver enzyme tests to determine which mothers had active hepatitis B infections and may require treatment themselves. However, we referred every mother who tested positive for HBsAg to a competent physician for consultation.

CONCLUSIONS

There is a high prevalence of Hepatitis B infection among pregnant women attending antenatal care in Gulu and Lacor Hospitals. A high proportion of the HBsAg positive mothers are also HBeAg positive and may be at an increased risk of transmitting HBV infection to their unborn babies. The pregnant women age ≤ 20 years are almost three times more likely to have Hepatitis B infection compared to the older women. There is need for screening of adolescent girls for Hepatitis B, and an urgent program on immunizing babies born to mothers who are HBsAg positive in order to reduce the risk of vertical transmission to their unborn babies, reduce the future population risks of developing liver complications related to HBV infections including hepatocellular carcinoma.

For peer review only - http://bmjopen.bmj.com/site/about/guidelines.xhtml

Conflict of interest

We have read and understood BMJ policy on declaration of interests and declare that we have no conflict of interest regarding the publication of this article.

Authors' contributions

PB, EO and ADM participated in study design and drafting manuscript. CO participated in data collection. PB and EO analyzed the data. ADM edited and reviewed the final version of manuscript for important intellectual content and consistency. All authors read and approved the final manuscript.

Acknowledgements

The authors are grateful to the participants for their cooperation and without whom there would have been no data for this study. The authors are indebted to the laboratory staff particularly Ms Peace Amito who prepared the standard operating procedures for laboratory tests. The authors are also thankful to the research assistants who diligently counseled participants and collected data. This work would have been incomplete without the cooperation of the administrators of the two study hospitals to whom the authors are grateful.

Funding

This work was supported by **Training Health Researchers into Vocational Excellence in East Africa (THRiVE)**, grant number 087540 funded by the Wellcome Trust. Its contents are solely the responsibility of the authors and do not necessarily represent the official views of the supporting offices.

Data Sharing Statement

No additional data available

For peer review only

Exclusive Licence

“The Corresponding Author has the right to grant on behalf of all authors and does grant on behalf of all authors, a worldwide licence to the Publishers and its licensees in perpetuity, in all forms, formats and media (whether known now or created in the future), to i) publish, reproduce, distribute, display and store the Contribution, ii) translate the Contribution into other languages, create adaptations, reprints, include within collections and create summaries, extracts and/or, abstracts of the Contribution, iii) create any other derivative work(s) based on the Contribution, iv) to exploit all subsidiary rights in the Contribution, v) the inclusion of electronic links from the Contribution to third party material where-ever it may be located; and, vi) licence any third party to do any or all of the above”.

For peer review only

For peer review only - http://bmjopen.bmj.com/site/about/guidelines.xhtml

References

1. Ching-Lung Lai, Man-Fung Yuen. Chronic Hepatitis B — New Goals, New Treatment. *N. Engl. J. Med.,* 2008;359(23):2488-91.

2. Ott JJ, Stevens GA, Wiersma ST. The risk of perinatal Hepatitis B virus transmission: hepatitis B e antigen (HBeAg) prevalence estimates for all world regions. *BMC Infectious Diseases* 2012;12(131).

3. Stevens CE, Toy PT, Tong MJ, Taylor PE, Vyas GN, Nair PV, et al. Hepatitis B Virus transmission in the United States, Prevention by Passive-Active Ummunization. *JAMA* 1985;253(12).

4. Tassopoulos NC, Papaevangelou GJ, Sjorgen MH, Roumeliotou-Karayanis A, Gerin JL p, RH. Natural History of acute Hepatitis B surface antigen positive hepatitis in Greek adults. *Gastroenterology* 1987;92(June).

5. Beasley PR. Hepatitis B virus as the etiologic agent in hepatocellular carcinoma, epidemiologic considerations. *Hepatology* 1982;Suppl:21S-26S.

6. Alter MJ. Epidemiology of Hepatitis B in Europe and worldwide. *J Hepatol* 2003;Suppl(1):S64-9.

7. WHO. Introduction of hepatitis B vaccine into childhood immunization services: Management guidelines, including information for health workers and parents. *Department of Vaccines and Biologicals*. Geneva: World Health Organisation, 2001.

8. US Preventive Services Task Force. Screening for Hepatitis B Virus Infection in Pregnancy: U.S Preventive Task Force Reaffirmation Recommendation Statement. *Ann Intern Med* 2009;150:869-73.

9. Ministry of Health Republic of Uganda. Annual Health Sector Performance Report 2007-2008,. In: Health, editor: MoH, 2008.

10. Lavanchy D. Hepatitis B virus epidemiology, disease burden, treatment, and current and emerging prevention and control measures. *Journal of Viral hepatitis* 2004;11:97-107.

11. Andre FE, Zuckerman AJ. Protective efficacy of Hepatitis B vaccines in neonates. *Journal of Medical Virology* 1994;44:144-41.

12. Mbaawuaga E. M, Enenebeaku M.N.O, Okopi J. A, J.G Damen. Hepatitis B Virus (HBV) Infection among Pregnant Women in Makurdi, Nigeria. *African Journal of Biomedical Research* 2008;11(2008):155-59.

For peer review only

For peer review only - http://bmjopen.bmj.com/site/about/guidelines.xhtml

13. Rujumba J, Kwiringira J. Interface of culture, insecurity and HIV and AIDS: Lessons from displaced communities in Pader District, Northern Uganda. *Conflict and health* 2010;4:18.

14. Ministry of Health Uganda. Uganda HIV/AIDS Sero-behavioural Survey 2004-2005. Calverton Maryland, USA: Uganda Ministry of Health and ORC Macro, 2006:125-32.

15. Ochola E, Ocama P, Orach CG, Nankinga ZK, Kalyango JN, McFarland W, et al. High burden of Hepatitis B infection in Northern Uganda: results of a population-based survey. *BMC Public Health* 2013;13(727).

16. St. Mary's Hospital Lacor. *Lacor Hospital Annual Report Financial Year 2012/2013*. Gulu: Lacor Hospital, 2013.

17. Maria F. Pirillo, Luciana Bassani, Elena A.P. Germinario, Maria Grazia Mancini, Joseph Vyankandondera, Pius Okong, et al. Seroprevalence of Hepatitis B and C Viruses Among HIV-Infected Pregnant Women in Uganda and Rwanda. *Journal of Medical Virology* 2007;79(2007):1797–801.

18. Lara Stabinkski, Steven J Reynolds, Ponsiano Ocama, Oliver Laeyendecker, David Serwadda, Ron H G, et al. Hepatitis B virus and sexual behaviour in Rakai, Uganda. *J Med Virol* 2012;83(5):796-800.

19. Bwogi J, Braka F, Makumbi I, Mishra V, Bakamutumaho B, Nanyunja M, et al. Hepatitis B infection is highly endemic in Uganda: findings from a national serosurvey. *Afr Health Sci* 2009;9(2):98–108.

20. Mansour W, Malick F-ZF, Sidiya A, Ishaga E. Prevalence, Risk factors, and Molecular Epidemiology of Hepatitis B and Hepatitis Delta Virus in pregnant women and in patients in Mauritania. *Journal of Medical Virology* 2012;84(April):1186-98.

21. Ministry of Health Uganda. Uganda AIDS Indicator Survey 2011: MoH, 2012.

22. Råssjö EB, Mirembe F ED. Self reported sexual behaviour among adolescent girls in Uganda: reliability of data debated. *African Health Sciences* 2011;11(3):383-89.

23. Giles ML, Visvanathan K, Lewin SR, Sasadeuz J. Chronic Hepatitis B Infection and Pregnancy. *Obstetrical and Gynaecological Survey* 2012;67(1):37-44.

24. Dwivedi M, Misra SP, Mishra V, Pandey A, Pant S, Singh R, et al. Seroprevalence of Hepatitis B infection during pregnancy and risk of perinatal transmission. *Indian journal of gastroenterology* 2011;30(2):66-71.

25. Phillippe R, Alassane D, Chiekh B, Souleymane M, D F. Perinatal Transmission of Hepatitis B virus in Senegal, West Africa. *Journal of Viral Immunology* 1993;6(1):65-73.

For peer review only

26. Khamduang W, Ngo-giang-huong N, Gaudy-graffin C, Jourdain G, Suwankornsakul W, Jarupanich T. Prevalence, Risk Factors, and Impact of Isolated Antibody to Hepatitis B core antigen and Occult Hepatitis B virus infection in HIV-1-Infected Pregnant Women. *Clinical Infectious Diseases* 2013:1-9.

For peer review only

For peer review only - http://bmjopen.bmj.com/site/about/guidelines.xhtml

STROBE 2007 (v4) Statement—Checklist of items that should be included in reports of *cross-sectional studies*

Section/Topic	Item #	Recommendation	Reported on page #
**Title and abstract**	1	(*a*) Indicate the study's design with a commonly used term in the title or the abstract	1
		(*b*) Provide in the abstract an informative and balanced summary of what was done and what was found	2
**Introduction**			**4**
Background/rationale	2	Explain the scientific background and rationale for the investigation being reported	4
Objectives	3	State specific objectives, including any prespecified hypotheses	5
**Methods**			**6**
Study design	4	Present key elements of study design early in the paper	6
Setting	5	Describe the setting, locations, and relevant dates, including periods of recruitment, exposure, follow-up, and data collection	6
Participants	6	(*a*) Give the eligibility criteria, and the sources and methods of selection of participants	6
Variables	7	Clearly define all outcomes, exposures, predictors, potential confounders, and effect modifiers. Give diagnostic criteria, if applicable	7
Data sources/ measurement	8*	For each variable of interest, give sources of data and details of methods of assessment (measurement). Describe comparability of assessment methods if there is more than one group	7
Bias	9	Describe any efforts to address potential sources of bias	
Study size	10	Explain how the study size was arrived at	6
Quantitative variables	11	Explain how quantitative variables were handled in the analyses. If applicable, describe which groupings were chosen and why	
Statistical methods	12	(*a*) Describe all statistical methods, including those used to control for confounding	9
		(*b*) Describe any methods used to examine subgroups and interactions	
		(*c*) Explain how missing data were addressed	
		(*d*) If applicable, describe analytical methods taking account of sampling strategy	
		(*e*) Describe any sensitivity analyses	
**Results**			10

For peer review only

Participants	13*	(a) Report numbers of individuals at each stage of study—eg numbers potentially eligible, examined for eligibility, confirmed eligible, included in the study, completing follow-up, and analysed	10
		(b) Give reasons for non-participation at each stage	
		(c) Consider use of a flow diagram	
Descriptive data	14*	(a) Give characteristics of study participants (eg demographic, clinical, social) and information on exposures and potential confounders	10
		(b) Indicate number of participants with missing data for each variable of interest	
Outcome data	15*	Report numbers of outcome events or summary measures	12
Main results	16	(*a*) Give unadjusted estimates and, if applicable, confounder-adjusted estimates and their precision (eg, 95% confidence interval). Make clear which confounders were adjusted for and why they were included	13
		(*b*) Report category boundaries when continuous variables were categorized	
		(*c*) If relevant, consider translating estimates of relative risk into absolute risk for a meaningful time period	
Other analyses	17	Report other analyses done—eg analyses of subgroups and interactions, and sensitivity analyses	14
**Discussion**			16
Key results	18	Summarise key results with reference to study objectives	16
Limitations	19	Discuss limitations of the study, taking into account sources of potential bias or imprecision. Discuss both direction and magnitude of any potential bias	19
Interpretation	20	Give a cautious overall interpretation of results considering objectives, limitations, multiplicity of analyses, results from similar studies, and other relevant evidence	17
Generalisability	21	Discuss the generalisability (external validity) of the study results	18
**Other information**			
Funding	22	Give the source of funding and the role of the funders for the present study and, if applicable, for the original study on which the present article is based	20

*Give information separately for cases and controls in case-control studies and, if applicable, for exposed and unexposed groups in cohort and cross-sectional studies.

Note: An Explanation and Elaboration article discusses each checklist item and gives methodological background and published examples of transparent reporting. The STROBE checklist is best used in conjunction with this article (freely available on the Web sites of PLoS Medicine at http://www.plosmedicine.org/, Annals of Internal Medicine at http://www.annals.org/, and Epidemiology at http://www.epidem.com/). Information on the STROBE Initiative is available at www.strobe-statement.org.

For peer review only

BMJ Open

High prevalence of Hepatitis B virus infection among pregnant women attending antenatal care: a cross-sectional study in two hospitals in northern Uganda

For peer review only

Journal:	*BMJ Open*
Manuscript ID:	bmjopen-2014-005889.R1
Article Type:	Research
Date Submitted by the Author:	19-Aug-2014
Complete List of Authors:	Bayo, Pontius; St. Mary's Hospital Lacor, Obstetrics and Gynecology<br>Ochola, Emmanuel; Gulu University Faculty of Medicine, Public Health<br>Oleo, Carolyne; Makerere University College of Health Sciences, School of Medicine, Obstetrics & Gynecology<br>Mwaka, Amos; Mulago Hospital/Makerere University, Department of Internal Medicine
**Primary Subject Heading**:	Infectious diseases
Secondary Subject Heading:	Obstetrics and gynaecology
Keywords:	Infection control < INFECTIOUS DISEASES, Prenatal diagnosis < OBSTETRICS, PREVENTIVE MEDICINE, Hepatobiliary tumours < ONCOLOGY

SCHOLARONE™
Manuscripts

High prevalence of Hepatitis B virus infection among pregnant women attending antenatal care: a cross-sectional study in two hospitals in northern Uganda

Corresponding author: Pontius Bayo, St. Mary's Hospital Lacor, P.O. Box 180, Gulu, Uganda.

Email: pontiusby@gmail.com Tel. +256 782 1424 35

Co- authors:

Emmanuel Ochola[1,2], Caroline Oleo[2,3], Amos Deogratius Mwaka[3]

1. St. Mary's Hospital Lacor, P.O. Box 180, Gulu, Uganda. 2. Gulu University Medical School, P.O. Box 166, Gulu, Uganda. 3. Makerere University College of Health Sciences, School of Medicine, P. O. Box 7072, Kampala, Uganda.

Key words: Hepatitis B virus, Prevalence, Pregnant women, Northern Uganda

Word count: 3741.

For peer review only

ABSTRACT

Objective: To determine prevalence of hepatitis B viral (HBV) infection and Hepatitis B e antigen (HBeAg) positivity among pregnant women attending antenatal clinics in two referral hospitals in northern Uganda.

Design: Cross-sectional observational study.

Setting: Two tertiary hospitals in a post-conflict region in a low-income country.

Participants: Randomly selected 402 pregnant women attending routine antenatal care in two referral hospitals. Five women withdrew consent for personal reasons. Data was analyzed for 397 participants.

Primary outcome: Hepatitis B surface antigen (HBsAg) positivity.

Results: Of 397 pregnant women aged 13-43 years, 96.2% were married or cohabiting. 47 (11.8%) tested positive for hepatitis B surface antigen (HBsAg); of these, 7 (14.9 %) were HBeAg positive. The highest HBsAg positivity rate was seen in women aged 20 years or less (20%) compared to those above 20 years old (8.7%), aOR = 2.54 (95% CI: 1.31 - 4.90). However, there was no statistically significant difference between women with positive HBsAg and those with negative tests results with respect to median values of liver enzymes, hemoglobin level, absolute neutrophil counts and white blood cells (WBC) counts. HIV positivity, scarification, and number of sexual partners were not predictive of HBV positivity.

Conclusion: One in eight pregnant women attending antenatal care in the two study hospitals has evidence of Hepatitis B infection. A significant number of these mothers are HBe Ag positive and may be at an increased risk of transmitting hepatitis B infection to their unborn

For peer review only - http://bmjopen.bmj.com/site/about/guidelines.xhtml

babies. We suggest that all pregnant women attending ante natal care be tested for hepatitis B viral infection; exposed babies need to receive HBV vaccines at birth.

For peer review only

Strengths and limitations of this study

- In this study we have evaluated the prevalence of a sexually transmitted viral infection, a risk factor for hepatocellular carcinoma in a population exposed to no condom sexual intercourse in a post-conflict region with high rates of HIV infection, another surrogate marker for sexually transmitted infections.
- We also investigated the prevalence of hepatitis B e antigen, a surrogate measure of the risk of vertical transmission of hepatitis B infection. This is important in determining need for immediate vaccinations of babies after birth.
- Findings from this study may inform policy on routine testing of pregnant women and immunization of HBV exposed babies at birth in addition to the current practice of using combined vaccine at six weeks.
- The study had some limitations; it was hospital-based and included a selected population of women with exposure to no condom sexual intercourse and therefore at high risk of sexually transmitted infections including hepatitis B virus and HIV. In addition we could not demonstrate evidence for chronicity of hepatitis B infections because we did not perform tests for Hepatitis B core antibodies (anti-HBc) and HBV DNA because of logistical reasons.

For peer review only - http://bmjopen.bmj.com/site/about/guidelines.xhtml

INTRODUCTION

Four hundred million people in the world are living with chronic hepatitis B virus (HBV) infection.[1] The majority of these individuals acquired the infection during the peri-natal period and early childhood.[2] The risk of becoming a chronic Hepatitis B infection carrier is 95% for infections acquired during the perinatal period[3] compared to only 5% for those acquired during adulthood.[4] Up to 50% of HBV carriers die of complications including liver cirrhosis and hepatocellular carcinoma.[5]

Pregnant mothers who test positive for both Hepatitis B surface antigen (HBsAg) and Hepatitis B e antigen (HBeAg) have 70 - 90% risk of transmitting infection to their new borne and about 10 - 40% if they test positive for only HBsAg.[5 6] Therefore, pregnant women should be routinely screened for HBsAg and Hepatitis B vaccine administered at birth to the infants whose mothers test positive.[7 8] However, this is not the practice in Uganda.

The Uganda National Expanded Program on Immunizations (UNEPI) scaled-up childhood immunizations in 2002,[9] incorporating the hepatitis B vaccine into a combination vaccine whose first dose is administered at six weeks of age. The six weeks window both limits the efficacy of the vaccine in the prevention of vertical transmission and also allows for the potential transmission of HBV through close contacts.[7] The most effective method of preventing HBV infection is through immunization which offers over 95% protection against the development of chronic infection.[10] Such immunization should be done at birth for exposed infants. There is no evidence of protection against perinatal transmission if the first dose of vaccine is given more than seven days after birth.[11]

In Nigeria, the prevalence of HBV infection among pregnant women was 11% with HbeAg positivity of 33%.[12] In northern Uganda, there is limited knowledge on the prevalence of

For peer review only - http://bmjopen.bmj.com/site/about/guidelines.xhtml

hepatitis B infection among pregnant women. The civil war in this region between the government of Uganda and the Lord's resistance army from the late 1980's up to 2006 led to the displacement of as many as 1.7 million people from their homes into internally displaced persons (IDP) camps.[13] These camps were crowded, traditional and social structures were disrupted and sexually transmitted infections such as HBV seemed to have increased. The Uganda HIV sero-behavioral survey of 2004/2005 estimated the prevalence of hepatitis B in northern Uganda to be between 18.4% and 24.3%, much higher than the national average of 10%,[14] while in a recent community-based study in Gulu municipality the prevalence of HBV in the general population was estimated at 17.6%.[15]

In this study we report the prevalence of HBV infection among pregnant women attending antenatal care (ANC) at St. Mary's Hospital Lacor (Lacor) and Gulu Regional referral Hospital using HBsAg test. We also report HBeAg positivity, a surrogate measure of infectivity among those women who tested positive for HBsAg and describe the factors associated with HBV infection among these women, with possible implications for testing of pregnant mothers, as well as vaccination of HBV exposed neonates.

For peer review only

METHODS

Study design and setting

This was a cross-sectional study at Lacor and Gulu regional referral Hospitals. The two hospitals are both in Gulu district in northern Uganda. Lacor hospital is 6 kilometers west of Gulu town; it is a 482 bed capacity teaching Hospital[16] and a sentinel site for infectious disease surveillance in northern Uganda, and has a laboratory with capacity for the separation and storage of frozen plasma. Lacor Hospital antenatal clinic (ANC) is visited by 50 - 80 pregnant women per day, Monday through Friday. Gulu regional referral Hospital on the other hand, is a 250-beds government owned referral facility located in the center of Gulu town;[16] the antenatal clinic in Gulu hospital is visited by about 40 - 60 pregnant women every working day.

Study population

We included pregnant women attending ante natal care at the two study hospitals during September, 2012 through January, 2013, whose gestation age was 28 weeks or more confirmed by clinical history and examination or obstetric ultra sound scan. We excluded women who had emergency conditions requiring urgent intervention. The two hospitals receive majority of pregnant women from Gulu district; however some women attend ante natal care in other private facilities in the town and health centers.

Sample size and sampling method

We used the Kish Leslie formula (1965) and a prevalence of HBsAg of 30% for sample size determination, to cater for the North-central Uganda prevalence of about 20%[14] and an additional 10% since pregnant women are engaged in unprotected sex, a known risk factor for sexually

For peer review only

transmitted infections compared to the general population.[17] To cater for possible incomplete responses, we added 10% of the calculated sample size, hence 402 participants were recruited.

Sampling procedures

Women were sampled on two working days a week in the two study hospitals; Lacor on Wednesdays and Fridays while in Gulu, sampling was done on Mondays and Thursdays. All eligible pregnant women attending ante natal care on the study days were verbally informed of the study immediately after routine ante natal care health education. We used systematic random sampling, selecting every 5th woman on the ante natal care waiting line.

Data collection procedures

At each study site, two midwives were trained for two days on study procedures, facts on HBV infections and transmissions, counseling, safety issues, sample collection and transportation as well as site testing for HBsAg.

Upon obtaining written informed consents, a questionnaire was administered to every selected woman to obtain socio-demographic information including maternal age, gestation age, gravidity, occupation, marital status and highest level of education. Other information on risk factors for transmission of HBV including history of previous blood transfusions, and history of scarification were also obtained. The women were then helped to immediately receive care from the clinic staffs.

Participants were informed that those who test positive for HBsAg would be called back to receive results of another test (HBeAg) to be done on their stored blood samples. They were also counseled about the hepatitis B vaccine that the study would provide to their infants at birth.

For peer review only - http://bmjopen.bmj.com/site/about/guidelines.xhtml

Plasma-derived hepatitis B vaccine was administered to infants born to HBsAg positive mothers within 12 hours of birth as recommended by World Health Organisation.[18] Each vaccine dose (0.5 ml) contained 10 pg of purified HBsAg.

Laboratory procedures

Trained research assistants provided pre-test counseling on hepatitis B viral and HIV infections. Five milliliters (5 mls) of blood was then drawn by veni-puncture from the cubital fossa under aseptic techniques. The blood samples were immediately put into portable cold boxes with ice packs. The research assistants immediately transported samples to the laboratories at study sites to test for HBsAg test (i.e. at Lacor and Gulu Hospital Laboratories). In the meantime, the women were helped to obtained ante natal care. Results were collected back by the research assistants who provided post-test counseling and released results to the participants on the same visit day. Blood samples positive for HBsAg from Gulu hospital were transported on same day to Lacor hospital laboratory and were frozen at -80°C and later transferred to MBN Clinical laboratories in the capital Kampala, for HBeAg testing.

Testing for HBsAg was done using the Infectious Diseases Enzyme Linked Immunosorbent Assay (ELISA) kits provided by Savyon Diagnostics Ltd, Ashdod, Israel, which has sensitivity of 99% and specificity of 96.7%. Testing for HbeAg was done using the Infectious Diseases ELISA - peroxidase conjugated kits, which have 100% sensitivity and 99.9% specificity, and inbuilt quality controls.

Samples from all the participants were tested for HIV, complete blood counts (CBC), liver alanine aminotransferase (ALT), aspartate aminotransferase (AST), alkaline phosphatase (ALP), using a SMAC auto-analyzer (Semi Micro Analyzer Computer, Technicon, USA). Complete

For peer review only

For peer review only - http://bmjopen.bmj.com/site/about/guidelines.xhtml

blood count was done with an automated analyzer, Humacount 60[TS]. HIV tests were performed using rapid assay for HIV antibody testing.

Data analysis

Data entered in Microsoft Excel was exported to STATA software version 12 for analysis. We described data using proportions, medians and interquartile range. Association between participant characteristics and HBsAg positivity was assessed using chi-square test (or Fishers' exact test as appropriate) for categorical predictors, or Wilcoxon rank sum test for the continuous laboratory parameters which were not normally distributed (tested using Shapiro Wilk test). Logistic regression was done to predictors of HBsAg positivity at multivariate level. A p-value of ≤ 0.05 was considered statistically significant in all statistical tests.

Ethical considerations

Each prospective participant received explanation about the study in their language of choice, mostly Acholi, the major Ugandan language spoken in the study region. They were provided with and given 20 minutes to study the IRC stamped consent forms in the local language; and thereafter, requested for their informed consents to participate in the study. Questionnaires were administered only after signed or thumb printed consents. All participants did not pay for tests done, and test results were provided to the women. All infants born to mothers positive for HBsAg received hepatitis B vaccines at the costs of the study team. Institutional ethical approval was received from Lacor hospital Ethics Review Committees (IRC), and the Uganda National Council of Science and Technology (UNCST), with permission to consider pregnant mothers under 18 years as emancipated minors capable of consenting.

For peer review only - http://bmjopen.bmj.com/site/about/guidelines.xhtml

RESULTS

Study participants

We approached 402 participants (200 from Lacor and 202 from Gulu Hospital). Five mothers withdrew consent; we therefore, included 397 participants in the analysis.

The median age of the participants was 24 years (range: 13-43 years). Regarding ethnicity, 89% (n=356) of the participants were of Acholi tribe. Up to 96.2% (n=382) of the women were either married or cohabiting; 71.8% (n=285) of the married women were in monogamous relationship (Table 1).

For peer review only

Table 1: Socio-demographic characteristics of 397 antenatal Hepatitis B study participants

Variable	Frequency, N=397	Percent (%)
**Age**		
≤ 20 years	110	27.7
>20 years	287	72.3
**Education**		
Informal education	30	7.6
Primary	191	48.0
Secondary	140	35.3
Tertiary	41	9.1
**Tribe**		
Acholi	356	89.7
Lango	17	4.3
Others*	24	6.0
**Occupation**		
Peasant	220	55.4
Professional	44	11.1
Other	133	33.5
**Marital status**		
Single	15	3.8
Married	160	40.3
Cohabiting	222	55.9
**Type of marriage**		
Monogamous	285	71.8
Polygamous	112	28.2
**Parity**		
0	93	23.4
1-4	250	63.0
5+	54	13.6
**HIVstatus**		
Negative	360	90.7
Positive	37	9.3
**Scarification**		
Not Done	43	10.8
Done	354	89.2

*Other tribes include Madi, Baganda, Jalwo, Karimojong, Banyoro

For peer review only

For peer review only - http://bmjopen.bmj.com/site/about/guidelines.xhtml

Prevalence hepatitis B, HIV and HBeAg positivity

The overall prevalence of Hepatitis B surface antigen (HBsAg) positivity was 11.8%; the prevalence was 12.7% and 10.9% in Lacor and Gulu Hospitals respectively (Table 2). Hepatitis B e Antigen was positive in 7 of the 47 HBsAg positive women (14.9%).

Hepatitis B surface antigen positive mothers were significantly younger than the negative mothers (p = 0.002) (Table 2).

Antibody test for HIV infection was positive among 9.3% (n=37) of participants, but there was no statistically significant association between HIV infection status and Hepatitis B prevalence, OR 0.89 (CI 0.30-2.65, p=0.839).

For peer review only - http://bmjopen.bmj.com/site/about/guidelines.xhtml

Table 2: Association between participants' characteristics (socio-demographic and clinical) and HBsAg positivity

Variable	Hepatitis B infection (HBsAg result)				
	N (+ve)	N (–ve)	% +ve	Crude OR (CI)	P value
**Overall prevalence**	47*	350	11.8	--	--
**Age**					
≤ 20 years	22	88	20	2.62 (1.41-4.89)	0.002**
>20 years	25	262	8.7	Ref	
**Education**					
Informal	6	24	20	ref	
Primary	17	174	8.9	0.39 (0.14-1.09)	0.070
Secondary	20	120	14.3	0.67 (0.24-1.83)	0.432
Tertiary	4	32	11.8	0.50 (0.13-1.97)	0.322
**Marital status**					
Not married	30	207	12.7	ref	
Married	17	143	10.6	0.82 (0.44-1.54)	0.539
**Parity**					
0	13	80	14.0	ref	
1-4	29	221	11.6	0.81 (0.40-1.63)	0.551
5+	5	49	9.3	0.63 (0.21-1.87)	0.403
**HIVstatus**					
Negative	43	317	11.94	ref	
Positive	4	33	10.81	0.89 (0.30-2.65)	0.839
**Scarification**					
Not Done	5	38	10.6	ref	
Done	42	312	11.2	1.02 (0.38-2.74)	0.934
**History of Blood transfusion**					
No	44	339	11.5	ref	
Yes	3	11	21.43	2.10(0.56-7.82	0.268
**No. of sexual partners last 2yrs**					
1	43	334	11.4	ref	
2	4	16	20.0	1.93 (0.62-6.08)	0.254

*Of the 47 with HBsAg positive result, 7 (14.9%) were found Hepatitis B e Antigen positive

**At Multivariate level, only age was a significant predictor of HBsAg positivity, at aOR=2.54 (1.31-4.90); p value 0.006.

For peer review only - http://bmjopen.bmj.com/site/about/guidelines.xhtml

Hepatitis B risk factors

Risk factors including history of scarification, number of sexual partners, history of blood transfusion or polygamy had no statistically significant relationship with HBsAg positivity (table 2). The liver function tests and complete blood cell counts were similar in both HBs Ag positive and negative women. Majority of women had hemoglobin concentrations (Hb) and platelets counts within normal ranges; these counts were not predictive of HBsAg positivity (table 3).

Table 3: Association between participants' Laboratory test results and HBsAg positivity

Variable (Median, IQR)	**HBsAg positive**	**HBsAg negative**	**p-value***
WBC	6400 (5180-8100)	6100 (5100-7300)	0.294
Lymphocytes count	1700 (1300-2100)	1575 (1250-1900)	0.117
Neutrophils count	3900 (1300-5100)	3900 (3040-4900)	0.619
Haemoglobin	12.2 (11.4-12.7)	11.9 (11.1-12.7)	0.357
ALP	284 (190-343)	259 (193-336)	0.452
AST	25 (17-27)	21 (13-29)	0.251

For peer review only

ALT	21 (12-30)	18 (11-26)	0.314

*The Mann Whitney test was used to test differences in the groups as all the variables were not normally distributed as tested by Shapiro Wilk test.

On multivariable analysis, women 20 years of age or younger were 2.5 folds more likely to test positive than those aged over 20 years; aOR 2.52, CI (1.31-4.90); p value 0.006) (Table 2 footnote).

For peer review only

For peer review only - http://bmjopen.bmj.com/site/about/guidelines.xhtml

DISCUSSION

This study highlights the high prevalence of Hepatitis B virus infection (11.8%) among pregnant women attending ante natal care in two hospitals in post-conflict northern Uganda. Although the prevalence of both HBV and HIV infections in this region exceeds those in most other regions of Uganda that have not experienced prolonged civil conflict and internment in camps, no causal relationship between HBV infection and civil conflict can be inferred from this findings from a cross-sectional study. We also found that about 15% of the HBsAg positive mothers were also HBeAg positive. The prevalence of HBV infection was higher among women aged 20 years or younger (20%) compared to the older women (8.7%). HIV infection among the study population was also high (9.3%). However there was no significant association between HIV infection and HBV infection among the pregnant women included in this study.

The prevalence of HBV infection among pregnant women in this study is consistent with findings from a study in Nigeria of a prevalence of 11%. The prevalence of HBeAg (33%) was however higher in the Nigerian study.[12] The majority of people who get HBV infection after neonatal period tend to clear the virus over time. The natural history of hepatitis B infection follows three phases; immune tolerant, immune active and immune inactive phases. During the immune active phase when the virus is actively replicating and HBV DNA is high, HBeAg becomes positive and the individual is at higher risk of transmitting the virus. In the immune inactive phase, the individual has cleared the virus and HBsAg from blood and becomes less or not infectious to others unless they revert to the immune active phase. The liver enzymes particularly ALT are normal during the immune tolerant and immune inactive phases. In our study the liver enzymes were largely within normal ranges and did not vary significantly

For peer review only - http://bmjopen.bmj.com/site/about/guidelines.xhtml

between HBsAg positive and negative pregnant mothers. This may mean that most of our mothers were in the immune intolerant or immune inactive phases of their infections. In the Nigerian study where prevalence of HBeAg was up to 33%. It is probable that the mothers were in the immune active phases and could have had recent infections or were reverting from immune inactive to active phases[19]. The finding in this study that 3 in 20 pregnant women with positive HBsAg are also HBeAg positive means that many unborn babies in northern Uganda are at even higher risk of infection with HBV. The infants of all these HBsAg positive mothers will need immediate vaccination with HBV vaccine upon delivery. This is, however, not the practice in Uganda and that means the risk of infection is not adequately minimized in these infants. Children who contract HBV infections from their mothers are more likely to develop chronic HBV infection and progress to liver complications associated with active HBV infection including cirrhosis and hepatocellular carcinoma.

To demonstrate a need for a specific affirmative program to reduce the incidence of complications from chronic HBV infections in this community, we discuss our findings in context of HBV infections in Uganda as a whole. Review of the sentinel surveillance data shows that the HBV infections prevalence in this study is higher than HBV infection prevalence among HIV positive pregnant women (4.9%) in central Uganda[20] and among HIV infected adult population (5%) in Rakai, south western Uganda[21]. The prevalence of HBV infection of 18 - 24% in the general population in northern Uganda is in fact higher than in most parts of Uganda, and higher among males than females,[14 22] and so the findings in this study for the pregnant population just mirrors the background female population prevalence in northern Uganda. In this study, the prevalence of HBV infection was higher among the younger compared to the older women. This is in variance to findings from a study in Mauritania where there was no significant

For peer review only

difference in the mean age of pregnant women who were HBsAg positive compared to those who were negative.[23] Our finding is however, similar to results from the Uganda national sero-behavioral survey in 2005 which showed a prevalence of 8.8% in the age group 15-19 years of age and increments with age[22] and in Rakai where positive HBsAg tests reached the highest at 8% among the age group 20-29 years of age.[21] The high prevalence of HBV infection among the younger age group in this study and in the general Ugandan population may be related to the relatively high vulnerability of the younger women to sexually transmitted infections.[24] In northern Uganda where people lived in the camps for more than 20 years, it is possible that these young women themselves acquired peri-natal HBV infections from their mothers who could have been exposed to sexually transmitted HBV during life in camps. A study by Råssjö et al, showed that females were more disposed to sexually transmitted infections (STIs) despite risky behavior being more common among males.[17] Biological and social factors including unemployment and little formal education contribute significantly to higher prevalence of STIs, including Hepatitis B, among adolescent girls. However, in our study there were no significant differences in employment status, education levels, marital status and number of sexual partners in the previous two years among HBsAg positive participants and those who were negative. The risk of vertical transmission of HBV infection to the unborn child may be related to the effect of HBV infection on the mother; how she responds to the infection, the timing of the infection with respect to the current pregnancy and the immune status of the mother as well as the levels of HBV DNA.[25] The probability of vertical infection is however, much increased when the mother is also positive for HBeAg.[3] In one study, vertical transmission was seen in 65% of babies born to mothers who were positive for HBeAg compared to 9.1% for babies born to mothers who were negative for HBeAg.[26] In Senegal, out of 21 infants born to HBsAg positive

For peer review only - http://bmjopen.bmj.com/site/about/guidelines.xhtml

mothers 11 were HBsAg positive at birth; and at 6 -7 months, five of these were still strongly HBsAg positive and developed antibodies to HBsAg, HBcAg or HBeAg.[27] At present, pregnant women in Uganda are not routinely screened for HBsAg, and the exposed newborns are not immunized at birth against HBV infection. This high prevalence rate of HBsAg positivity among asymptomatic pregnant women in our study shows that there are many infants born who are at high risk of becoming chronic Hepatitis B carriers and dying of chronic liver disease at young adult age in future.

This study is not without limitation. We did not test for anti-HBc and HBV DNA; and so there could have been HBsAg negative individuals with isolated anti-HBc and occult HBV infection. However, a recent study among HIV infected pregnant women showed that pregnant women with isolated anti-HBc and occult HBV infection have very low HBV DNA levels and are thus at very low risk to transmit HBV to their infants.[28] We also did not perform high resolution abdominal ultrasound scans nor did we carry out serial liver enzyme tests to determine which mothers had active hepatitis B infections and may require treatment themselves. However, we referred every mother who tested positive for HBsAg to a competent physician for consultation.

IMPLICATIONS AND RECOMMENDATIONS

Government and development partners in health need to pay special attention to the high prevalence of infection in this region in order to reduce the cost of care of chronic liver diseases including hepatocellular carcinoma in the future. There is need to urgently introduce routine screening for HBV infection during pregnancy and provide vaccination at birth for the exposed infants in order to reduce incidences of peri-natal infections with HBV.

For peer review only

We recommend further studies to better characterize the pattern of HBV infections among the younger age groups 18 – 25 years. Results of such studies might provide guidance on appropriate methods of interventions to reduce incidence and prevalence of HBV among these younger populations.

CONCLUSIONS

There is a high prevalence of Hepatitis B infection among pregnant women attending antenatal care in Gulu and Lacor Hospitals. A high proportion of the HBsAg positive mothers are also HBeAg positive and may be at an increased risk of transmitting HBV infection to their unborn babies. These babies are at high risk of becoming chronic carriers of HBV infections and subsequently increasing the population pool of the virus.

For peer review only

For peer review only - http://bmjopen.bmj.com/site/about/guidelines.xhtml

Acknowledgements

The authors are grateful to the participants for their participation and cooperation. The authors are indebted to the laboratory staff particularly Ms Peace Amito who prepared the standard operating procedures for laboratory tests. The authors are also thankful to the research assistants who diligently counseled participants and collected data. This work would have been incomplete without the cooperation of the administrators of the two study hospitals to whom the authors are grateful

Funding

This work was supported by **Training Health Researchers into Vocational Excellence in East Africa (THRiVE)**, grant number 087540 funded by the Wellcome Trust. Its contents are solely the responsibility of the authors and do not necessarily represent the official views of the supporting offices.

Authors' contributions

PB, EO and ADM participated in study design and drafting manuscript. CO participated in data collection. PB and EO analyzed the data. ADM edited and reviewed the final version of manuscript for important intellectual content and consistency. All authors read and approved the final manuscript

Conflict of interest

We have read and understood BMJ policy on declaration of interests and declare that we have no conflict of interest regarding the publication of this article.

Data Sharing Statement

For peer review only

No additional data available

Exclusive Licence

"The Corresponding Author has the right to grant on behalf of all authors and does grant on behalf of all authors, a worldwide licence to the Publishers and its licensees in perpetuity, in all forms, formats and media (whether known now or created in the future), to i) publish, reproduce, distribute, display and store the Contribution, ii) translate the Contribution into other languages, create adaptations, reprints, include within collections and create summaries, extracts and/or, abstracts of the Contribution, iii) create any other derivative work(s) based on the Contribution, iv) to exploit all subsidiary rights in the Contribution, v) the inclusion of electronic links from the Contribution to third party material where-ever it may be located; and, vi) licence any third party to do any or all of the above".

For peer review only

References

1. Ching-Lung Lai, Man-Fung Yuen. Chronic Hepatitis B — New Goals, New Treatment. *N. Engl. J. Med.,* 2008;359(23):2488-2491.

2. Ott JJ, Stevens GA, Wiersma ST. The risk of perinatal Hepatitis B virus transmission: hepatitis B e antigen (HBeAg) prevalence estimates for all world regions. *BMC Infectious Diseases* 2012;12(131).

3. Stevens CE, Toy PT, Tong MJ, et al. Hepatitis B Virus transmission in the United States, Prevention by Passive-Active Ummunization. *JAMA* 1985;253(12).

4. Tassopoulos NC, Papaevangelou GJ, Sjorgen MH, et al. Natural History of acute Hepatitis B surface antigen positive hepatitis in Greek adults. *Gastroenterology* 1987;92(June).

5. Beasley PR. Hepatitis B virus as the etiologic agent in hepatocellular carcinoma, epidemiologic considerations. *Hepatology* 1982;Suppl:21S-26S.

6. Alter MJ. Epidemiology of Hepatitis B in Europe and worldwide. *J Hepatol* 2003;Suppl(1):S64-9.

7. WHO. Introduction of hepatitis B vaccine into childhood immunization services: Management guidelines, including information for health workers and parents. *Department of Vaccines and Biologicals*. Geneva: World Health Organisation, 2001.

8. US Preventive Services Task Force. Screening for Hepatitis B Virus Infection in Pregnancy: U.S Preventive Task Force Reaffirmation Recommendation Statement. *Ann Intern Med* 2009;150:869-73.

9. Ministry of Health Republic of Uganda. Annual Health Sector Performance Report 2007-2008,. In: Health, editor: MoH, 2008.

10. Lavanchy D. Hepatitis B virus epidemiology, disease burden, treatment, and current and emerging prevention and control measures. *Journal of Viral hepatitis* 2004;11:97-107.

11. Andre FE, Zuckerman AJ. Protective efficacy of Hepatitis B vaccines in neonates. *Journal of Medical Virology* 1994;44:144-141.

12. Mbaawuaga E. M, Enenebeaku M.N.O, Okopi J. A, et al. Hepatitis B Virus (HBV) Infection among Pregnant Women in Makurdi, Nigeria. *African Journal of Biomedical Research* 2008;11(2008):155-159.

13. Rujumba J, Kwiringira J. Interface of culture, insecurity and HIV and AIDS: Lessons from displaced communities in Pader District, Northern Uganda. *Confl Health* 2010;4:18.

For peer review only - http://bmjopen.bmj.com/site/about/guidelines.xhtml

14. Ministry of Health Uganda. Uganda HIV/AIDS Sero-behavioural Survey 2004-2005. Calverton Maryland, USA: Uganda Ministry of Health and ORC Macro, 2006:125-132.

15. Ochola E, Ocama P, Orach CG, et al. High burden of Hepatitis B infection in Northern Uganda: results of a population-based survey. *BMC Public Health* 2013;13(727).

16. St. Mary's Hospital Lacor. *Lacor Hospital Annual Report Financial Year 2012/2013*. Gulu: Lacor Hospital, 2013.

17. Råssjö EB, Mirembe F ED. Self reported sexual behaviour among adolescent girls in Uganda: reliability of data debated. *African Health Sciences* 2011;11(3):383-389.

18. WHO. *Preventing Mother-to-Child Transmission of hepatitis B : Operational field guidelines for delivery of the birth dose of hepatitis B vaccine*. Manilla: WHO, 2006.

19. Sorrel MF, Belongia EA, Costa J, et al. National Institutes of Health Consensus Development Conference Statement: Management of Hepatitis B. *Ann Intern Med* 2009;150(2):104-10.

20. Maria F. Pirillo, Luciana Bassani, Elena A.P. et al.. Seroprevalence of Hepatitis B and C Viruses Among HIV-Infected Pregnant Women in Uganda and Rwanda. *Journal of Medical Virology* 2007;79(2007):1797–1801.

21. Lara Stabinkski, Steven J Reynolds, Ponsiano Ocama, et al. Hepatitis B virus and sexual behaviour in Rakai, Uganda. *J Med Virol* 2012;83(5):796-800.

22. Bwogi J, Braka F, Makumbi I, et al. Hepatitis B infection is highly endemic in Uganda: findings from a national serosurvey. *Afr Health Sci* 2009;9(2):98–108.

23. Mansour W, Malick F-ZF, Sidiya A, et al. Risk factors, and Molecular Epidemiology of Hepatitis B and Hepatitis Delta Virus in pregnant women and in patients in Mauritania. *Journal of Medical Virology* 2012;84(April):1186-1198.

24. Ministry of Health Uganda. Uganda AIDS Indicator Survey 2011: MoH, 2012.

25. Giles ML, Visvanathan K, Lewin SR, et al.Chronic Hepatitis B Infection and Pregnancy. *Obstetrical and Gynaecological Survey* 2012;67(1):37-44.

26. Dwivedi M, Misra SP, Mishra V, et al. Seroprevalence of Hepatitis B infection during pregnancy and risk of perinatal transmission. *Indian journal of gastroenterology* 2011;30(2):66-71.

27. Phillippe R, Alassane D, Chiekh B, et al. Perinatal Transmission of Hepatitis B virus in Senegal, West Africa. *Journal of Viral Immunology* 1993;6(1):65-73.

For peer review only

28. Khamduang W, Ngo-giang-huong N, Gaudy-graffin C, et al. Prevalence, Risk Factors, and Impact of Isolated Antibody to Hepatitis B core antigen and Occult Hepatitis B virus infection in HIV-1-Infected Pregnant Women. *Clinical Infectious Diseases* 2013:1-9.

For peer review only

For peer review only - http://bmjopen.bmj.com/site/about/guidelines.xhtml

High prevalence of Hepatitis B virus infection among pregnant women attending antenatal care: a cross-sectional study in two hospitals in northern Uganda

For peer review only

Corresponding author: Pontius Bayo, St. Mary's Hospital Lacor, P.O. Box 180, Gulu, Uganda.

Email: pontiusby@gmail.com Tel. +256 782 1424 35

Co- authors:

Emmanuel Ochola[1,2], Caroline Oleo[2,3], Amos Deogratius Mwaka[3]

1. St. Mary's Hospital Lacor, P.O. Box 180, Gulu, Uganda. 2. Gulu University Medical School, P.O. Box 166, Gulu, Uganda. 3. Makerere University College of Health Sciences, School of Medicine, P. O. Box 7072, Kampala, Uganda.

Key words: Hepatitis B virus, Prevalence, Pregnant women, Northern Uganda

Word count: ~~3,452~~3741.

ABSTRACT

Objective: To determine prevalence of hepatitis B viral (HBV) infection and Hepatitis B e antigen (HBeAg) positivity among pregnant women attending antenatal clinics in two referral hospitals in northern Uganda.

Design: Cross-sectional observational study.

Setting: Two ~~T~~tertiary hospitals in a post-conflict region in a low-income country.

Participants: Randomly selected 402 pregnant women attending routine antenatal care in two referral hospitals. F~~, however, f~~ive women withdrew consent for personal reasons. Data was analyzed for 397 participants.

Primary outcome: Hepatitis B surface antigen (HBsAg) positivity.

~~**Setting:** Tertiary hospitals in a post-conflict region in low-income country.~~

Results: Of 397 pregnant women aged 13-43 years, 96.2% were married or cohabiting.~~,~~ 47 (11.8%) tested positive for hepatitis B surface antigen (HBsAg); of these, 7 (14.9 %) were HBeAg positive. The highest HBsAg positivity rate was seen in women aged 20 years or less (20%) compared to those above 20 years old (8.7%), aOR = 2.54 (95%~~5~~ CI: 1.31 - 4.90). ~~and the difference was statistically significant(p = 0.006).~~ However, there was no statistically significant difference between women with positive HBsAg ~~versus~~ and those with negative tests results with respect to median values of liver enzymes ~~levels~~, hemoglobin level, absolute neutrophil counts and ~~mean~~ white blood cells (WBC) counts. HIV positivity, scarification, and number of sexual partners were not predictive of HBV positivity.

Conclusion: One in eight pregnant women attending antenatal care in the two study hospitals ha~~i~~s evidence of Hepatitis B infection~~ed~~. A significant number of these mothers are HBe Ag

For peer review only

positive and may be at an increased risk of transmitting hepatitis B infection to their unborn babies. We suggest that all pregnant women attending ante natal care be tested for hepatitis B viral infection; exposed babies need to receive HBV vaccines at birth. ~~Catch up vaccination to reduce burden of HBV infection in the community is recommended for adolescence and young adults who missed HBV immunizations in childhood.~~

For peer review only

For peer review only - http://bmjopen.bmj.com/site/about/guidelines.xhtml

For peer review only

For peer review only - http://bmjopen.bmj.com/site/about/guidelines.xhtml

Strengths and limitations of this study

- In t~~T~~his study ~~has~~ we have evaluated the prevalence of a sexually transmitted viral infection, a risk factor for hepatocellular carcinoma in a population exposed to no condom sexual intercourse in a post-conflict region with high rates of HIV infection, another surrogate marker for sexually transmitted infections.
- ~~This study has~~We also investigated the prevalence of hepatitis B e antigen ~~test~~, a surrogate measure of the risk of vertical transmission of hepatitis B infection. This is important in determining need for immediate vaccinations of babies after birth.
- ~~The~~ Findings from this study ~~provides~~ may inform~~useful information~~ ~~for~~ policy ~~formulation about~~on routine testing of pregnant women and immunization of HBV exposed babies at birth ~~rather than~~in addition to the current practice of using combined vaccine at six weeks.
- The study had some limitations; it was hospital-based and included a selected population of women with exposure to no condom sexual intercourse and therefore at high risk of ~~with respect to~~ sexually transmitted infections including hepatitis B virus and HIV. In addition we could not demonstrate evidence for chronicity of hepatitis B infections because we did not perform tests for Hepatitis B core antibodies (anti-HBc) and HBV DNA because of logistical reasons ~~to define chronic infections~~.

1

Formatted: Font: Bold

For peer review only

For peer review only - http://bmjopen.bmj.com/site/about/guidelines.xhtml

INTRODUCTION

Four hundred million people in the world are living with chronic hepatitis B virus (HBV) infection.[1] The majority of these individuals acquired the infection during the peri-natal period and early childhood.[2] The risk of becoming a chronic Hepatitis B infection carrier is 95% for infections acquired during the perinatal period[3] compared to only 5% for those acquired during adulthood.[4] Up to 50% of HBV carriers die of complications including liver cirrhosis and hepatocellular carcinoma.[5]

Pregnant mothers who test positive for both Hepatitis B surface antigen (HBsAg) and Hepatitis B e antigen (HBeAg) have 70 - 90% risk of transmitting infection to their new borne and about 10 - 40% if they test positive for only HBsAg.[5 6~~5, 6~~] Therefore, pregnant women should be routinely screened for HBsAg and Hepatitis B vaccine administered at birth to the infants whose mothers test positive.[7 8~~7, 8~~] However, this is not the practice in Uganda.

The Uganda National Expanded Program on Immunizations (UNEPI) scaled-up childhood immunizations in 2002,[9] incorporating the hepatitis B vaccine into a combination vaccine whose first dose is administered at six weeks of age. The six weeks window both limits the efficacy of the vaccine in the prevention of vertical transmission and also allows for the potential transmission of HBV through close contacts.[7] The most effective method of preventing HBV infection is through immunization which offers over 95% protection against the development of chronic infection.[10] Such immunization should be done at birth for exposed infants. There is no evidence of protection against perinatal transmission if the first dose of vaccine is given more than seven days after birth.[11]

In Nigeria, the prevalence of HBV infection among pregnant women was 11% with HbeAg positivity of 33%.[12] In northern Uganda, there is limited knowledge on the prevalence of

Field Code Changed
Field Code Changed
Field Code Changed
Field Code Changed
Field Code Changed
Field Code Changed
Formatted: Font color: Custom Color(RGB(35,31,32)), Superscript
Field Code Changed
Formatted: Superscript
Field Code Changed
Field Code Changed
Field Code Changed
Field Code Changed
Field Code Changed

For peer review only

hepatitis B infection among pregnant women. The civil war in this region between the government of Uganda and the Lord's resistance army from the late 1980's up to 2006 led to the displacement of as many as 1.7 million people from their homes into internally displaced persons (IDP) camps.[13] These camps were crowded, traditional and social structures were disrupted and sexually transmitted infections such as HBV seemed to have increased. The Uganda HIV sero-behavioral survey of 2004/2005 estimated the prevalence of hepatitis B in northern Uganda to be between 18.4% and 24.3%, much higher than the national average of 10%,[14] while in a recent community- -based study in Gulu municipality estimated the prevalence of HBV in the general population was estimated at 17.6%.[15]

Field Code Changed

Field Code Changed

In this study we report the prevalence of HBV infection among pregnant women attending antenatal care (ANC) at St. Mary's Hospital Lacor (Lacor) and Gulu Regional referral Hospital using HBsAg test. We also report HBeAg positivity, a surrogate measure of infectivity among those women who tested positive for HBsAg and describe the factors associated with HBV infection among these women, with possible implications for testing ~~and vaccination of adolescent girls~~of pregnant mothers, as well as vaccination of HBV exposed neonates.

For peer review only - http://bmjopen.bmj.com/site/about/guidelines.xhtml

For peer review only

METHODS

Study design and setting

This was a cross-sectional study at Lacor and Gulu regional referral Hospitals. The two hospitals are both in Gulu district in northern Uganda. Lacor hospital is 6 kilometers west of Gulu town; it is a 482 bed capacity teaching Hospital[16] and a sentinel site for infectious disease surveillance in northern Uganda, and has a laboratory with capacity for the separation and storage of frozen plasma. Lacor Hospital antenatal clinic (ANC) is visited by 50 - 80 pregnant women per day, Monday through Friday. Gulu regional referral Hospital on the other hand, is a 250-beds government owned referral facility located in the center of Gulu town;[16] the antenatal clinic in Gulu hospital is visited by about 40 - 60 pregnant women every working day.

Field Code Changed

Study population

We included pregnant women attending ante natal care at the two study hospitals during September, 2012 through January, 2013, whose~~.~~ ~~All pregnant women 28 weeks of~~ gestation age was 28 weeks or more confirmed by clinical history and examination or obstetric ultra sound scan ~~were included in the study~~. We excluded women who had emergency conditions requiring urgent intervention. The two hospitals receive majority of pregnant women from Gulu district; however some women attend ante natal care in other private facilities in the town and health centers.

Sample size and sampling method

We used the Kish Leslie formula (1965) and a prevalence of HBsAg ~~value~~ of 30% for sample size determination, to cater for the ~~in~~ North-central Uganda prevalence of about 2~~1~~0% ~~for~~

For peer review only

~~sample size determination.~~[14] ~~[14]~~ and ~~We allowed for~~ an additional 10% since pregnant women are engaged in unprotected sex, a known risk factor for ~~prevalence to account for variations due to the fact that pregnant women are a selected population with higher risk of~~ sexually transmitted infections compared to the general population.[17] ~~[14]~~ ~~(ref 22)~~ ~~A further 10% was added t~~To cater for possible incomplete responses, we ~~obtain~~added 10% ~~added 105~~ of the calculated sample size, ~~;~~ hence 402 participants were recruited.

Formatted: Superscript

Formatted: Superscript

Formatted: Superscript

Sampling procedures

Women were sampled on two working days a week in the two study hospitals; Lacor on Wednesdays and Fridays while in Gulu, sampling was done on Mondays and Thursdays. All eligible pregnant women attending ante natal care on the study days were verbally informed of the study immediately after ~~the~~ routine ante natal care health education. ~~They were informed that not all women will be selected because particular criteria of selection, not disclosed to them will be used.~~ We used s~~S~~ystematic random sampling, selecting every 5th woman on the ante natal care waiting line ~~was done~~. ~~All pregnant women 28 weeks of gestation age or more confirmed by clinical history and examination or obstetric ultra sound scan were included in the study. We excluded women who had emergency conditions requiring urgent intervention.~~

Data collection procedures

At each study site, two midwives were trained for two days on study procedures, facts on HBV infections and transmissions, counseling, safety issues, sample collection and transportation as well as site testing for HBsAg.

For peer review only - http://bmjopen.bmj.com/site/about/guidelines.xhtml

Upon obtaining written informed consents, a questionnaire was administered to every selected woman to obtain socio-demographic information including maternal age, gestation age, gravidity, occupation, marital status and highest level of education. Other information on risk factors for transmission of HBV including history of previous blood transfusions, and history of scarification were also obtained. The women were then helped to immediately receive care from the clinic staffs.

Participants were informed that those who test positive for HBsAg would be ~~informed and~~ called back to receive results of another test (HBeAg) to be done on their stored blood samples. They were also counseled about the hepatitis B vaccine that the study would provide to their infants at birth. Plasma-derived hepatitis B vaccine was administered to infants born to HBsAg positive mothers within 12 hours of birth~~.~~ as recommended by World Health Organisation~~(ref…)~~.[18] Each vaccine dose (0.5 ml) contained 10 pg of purified HBsAg.

Formatted: Superscript

Laboratory procedures

Trained research assistants provided pre-test counseling on hepatitis B viral and HIV infections. Five milliliters (5 mls) of blood was then drawn by veni-puncture from the cubital fossa under aseptic techniques. The blood samples were immediately put into portable cold boxes with ice packs. The research assistants immediately transported samples to the laboratories at study sites to test for HBsAg test (i.e. at Lacor and Gulu Hospital Laboratories). In the meantime, the women were helped to obtained ante natal care. Results were collected back by the research assistants who provided post-test counseling and released results to the participants on the same visit day. Blood samples positive for HBsAg from Gulu hospital were transported on same day to Lacor hospital laboratory and were frozen at -80°C and later transferred to MBN Clinical laboratories in the capital Kampala, for HBeAg testing.

For peer review only - http://bmjopen.bmj.com/site/about/guidelines.xhtml

Testing for HBsAg was done using the Infectious Diseases Enzyme Linked Immunosorbent Assay (ELISA) kits provided by Savyon Diagnostics Ltd, Ashdod, Israel, which has sensitivity of 99~~100~~% and specificity of 96~~9~~.7%. Testing for HbeAg was done using the Infectious Diseases ELISA - peroxidase conjugated kits, which have 100% sensitivity and 99.9% specificity, and inbuilt quality controls.

Samples from all the participants were tested for HIV, complete blood counts (CBC), liver alanine aminotransferase (ALT), aspartate aminotransferase (AST), alkaline phosphatase (ALP), using a SMAC auto-analyzer (Semi Micro Analyzer Computer, Technicon, USA). Complete blood count was done with an automated analyzer, Humacount 60[TS]. HIV tests were performed using rapid assay for HIV antibody testing.

Data analysis

Data entered in Microsoft Excel was exported to STATA software version 12 for analysis. We described data using ~~Descriptive data included~~ proportions, ~~means and~~ medians and interquartile range. ~~Statistical analysis was done using STATA software version 12.0.~~ Association between ~~demographic variables~~ participant characteristics and HBsAg positivity ~~and laboratory parameters were~~was assessed using chi-square test (or Fishers' exact test as appropriate) for categorical predictors, or Wilcoxon rank sum test for the continuous laboratory parameters which were not normally distributed (tested using Shapiro Wilk test).~~;~~ L~~l~~ogistic regression~~s were~~ was done~~carried~~ to ~~determine magnitudes of associations~~predictors of HBsAg positivity at multivariate level~~; differences in means were assessed using the student t-tests~~. A p-value of ≤0.05 was considered statistically significant in all statistical tests.

For peer review only

Ethical considerations

Each prospective participant received explanation about the study in their language of choice, mostly Acholi, the major Ugandan language spoken in the study region. They were provided with and given 20 minutes to study the IRC stamped consent forms in the local language; and thereafter, requested for their informed consents to participate in the study. Questionnaires were administered only after signed or thumb printed consents. All participants did not pay for tests done, and test results were provided to the women. All infants born to mothers positive for HBsAg received hepatitis B vaccines at the costs of the study team. Institutional ethical approval was ~~sought~~ received from Lacor hospital Ethics Review Committees (IRC), and the Uganda National Council of Science and Technology (UNCST), with permission to consider pregnant mothers under 18 years as emancipated minors capable of consenting.

For peer review only

For peer review only - http://bmjopen.bmj.com/site/about/guidelines.xhtml

RESULTS

Study participants

We approached 402 participants (200 from Lacor and 202 from Gulu Hospital). Five mothers withdrew consent; we therefore, included 397 participants in the analysis.

The median age of the participants was 24 years (range: 13-43 years). Regarding ethnicity, 89% (n=356) of the participants were of Acholi tribe. Up to 96.2% (n=382) of the women were either married or cohabiting; 71.8% (n=285) of the married women were in monogamous relationship (Table 1).

For peer review only - http://bmjopen.bmj.com/site/about/guidelines.xhtml

1 **Table 1: Socio-demographic characteristics of 397 antenatal Hepatitis B study participants**

Variable	Frequency, N=397	Percent (%)
**Age**		
≤ 20 years	110	27.7
>20 years	287	72.3
**Education**		
Informal education	30	7.6
Primary	191	48.0
Secondary	140	35.3
Tertiary	41	9.1
**Tribe**		
Acholi	356	89.7
Lango	17	4.3
Others*	24	6.0
**Occupation**		
Peasant	220	55.4
Professional	44	11.1
Other	133	33.5
**Marital status**		
Single	15	3.8
Married	160	40.3
Cohabiting	222	55.9
**Type of marriage**		
Monogamous	285	71.8
Polygamous	112	28.2
**Parity**		
0	93	23.4
1-4	250	63.0
5+	54	13.6
**HIVstatus**		
Negative	360	90.7
Positive	37	9.3
**Scarification**		
Not Done	43	10.8
Done	354	89.2

2

3 *Other tribes include Madi, Baganda, Jalwo, Karimojong, Banyoro

For peer review only

Prevalence hepatitis B, HIV and HBeAg positivity

The overall prevalence of Hepatitis B surface antigen (HBsAg) positivity was 11.8%; the prevalence was 12.7% and 10.9% in Lacor and Gulu Hospitals respectively (Table 2). Hepatitis B e Antigen was positive in 7 of the 47 HBsAg positive women (14.9%).

Hepatitis B surface antigen positive mothers were significantly younger than the negative mothers (p = 0.002) (Table 2).

~~The prevalence of~~ Antibody test for HIV infection was positive among 9.3% (n=37) of participants,~~the participants was 9.3%~~ but there was no statistically significant association between HIV infection status and ~~difference in~~ Hepatitis B prevalence~~ by HIV status~~, OR 0.89 (CI 0.30-2.65, ~~(~~p=0.839).

For peer review only

For peer review only - http://bmjopen.bmj.com/site/about/guidelines.xhtml

For peer review only

Table 2: Association between- participants' characteristics (socio-demographic and clinical) and HBsAg positivity~~socio-demographic and clinical characteristics of HBsAg positive mothers~~

Formatted Table

Variable ~~(n)~~	Hepatitis B infection (HBsAg result~~positive~~)				
	N (~~positive~~+ve)	N (–ve)	% +ve	Crude OR (CI)	P value
**Overall prevalence ~~(397)~~**	47*	350	11.8	--	--
**Age**					
≤ 20 years ~~(110)~~	22	88	20	2.62 (1.41-4.89)	0.002**
>20 years ~~(287)~~	25	262	8.7	Ref	

For peer review only - http://bmjopen.bmj.com/site/about/guidelines.xhtml

**Education**					
~~None~~ Informal~~(30)~~	6	24	20	ref	
Primary ~~(191)~~	17	174	8.9	0.39 (0.14-1.09)	0.070
Secondary~~(140)~~	20	120	14.3	0.67 (0.24-1.83)	0.432
Tertiary ~~(36)~~	4	32	11.8	0.50 (0.13-1.97)	0.322
**Marital status**					
Not married ~~(237)~~	30	207	12.7	ref	
Married	17	143	10.6	0.82 (0.44-1.54)	0.539
**Parity**					
0 ~~(93)~~	13	80	14.0	ref	
1-4 ~~(250)~~	29	221	11.6	0.81 (0.40-1.63)	0.551
5+ ~~(54)~~	5	49	9.3	0.63 (0.21-1.87)	0.403
**HIVstatus**					
Negative ~~(360)~~	43	317	11.94	ref	
Positive ~~(37)~~	4	33	10.81	0.89 (0.30-2.65)	0.839
**Scarification**					
Not Done ~~(43)~~	5	38	10.6	ref	
Done ~~(354)~~	42	312	11.2	1.02 (0.38-2.74)	0.934
**History of Blood transfusion**					
No ~~(383)~~	44	339	11.5	ref	
Yes ~~(14)~~	3	11	21.43	2.10(0.56-7.82	0.268
**No. of sexual partners last 2yrs**					
1 ~~(377)~~	43	334	11.4	ref	
2 ~~(20)~~	4	16	20.0	1.93 (0.62-6.08)	0.254

*Of the 47 with HBsAg positive result, 7 (14.9%) were found Hepatitis B e Antigen positive

**At Multivariate level, only ~~only~~ age was~~remained~~ a significant predictor of HBsAg positivity, at aOR=2.54 (1.31-4.90); p value 0.006.

Hepatitis B risk factors

~~Common r~~Risk factors ~~like~~ including history of scarification, number of sexual partners, history of blood transfusion or polygamy had no statistically significant relationship with HBsAg positivity (table 2). The liver function tests and complete blood cell counts were similar in both

For peer review only

HBs Ag positive and negative women. Majority of women had hemoglobin concentrations (Hb) and platelets counts within normal ranges; these counts were not predictive of HBsAg positivity (table 3).

Table 3: Association between participants' Laboratory test results ~~for~~ and HBsAg positivity~~e and negative mothers~~

Variable (Median, IQR)	HBsAg positive	HBsAg negative	p-value*
~~Mean~~ WBC ~~(SD)~~	~~6535.96 (2004.26)~~6400 (5180-8100)	~~6345.46 (1824.65~~6100 (5100-7300)	0.294~~506~~
~~Mean~~ Lymphocytes count ~~(SD)~~	~~1901.28~~1700 (1300~~725.07~~-2100)	~~1665.37~~1575 (1250-1900 ~~725.07~~)	0.117~~062~~
Neutrophils ~~(SD)~~ count	~~4247.45~~ 3900 (~~1628.74~~1300-5100)	~~4104.48~~3900 (~~1492.48~~3040-4900)	0.619~~940~~
Haemoglobin ~~(SD)~~	12.2 ~~12.07~~ (11.4-12.7~~0.93~~)	~~14.15~~11.9 (~~13~~11.1-12.7)~~.36)~~	0.357~~485~~

Formatted: Space After: 0 pt, Line spacing: single

Formatted Table

For peer review only - http://bmjopen.bmj.com/site/about/guidelines.xhtml

ALP	284 2.74 (190- 125.28343)	279.58259 (193- 336137.04)	0.45288 0
AST	22.9125 (17- 278.49)	22.49 21 (13- 2914.63)	0.25184 6
ALT	21.68 21 (12- 3011.75)	21.4418 (11- 2618.40)	0.31493 3

*The Mann Whitney test was used to test differences in the groups as all the variables were not normally distributed as tested by Shapiro Wilk test.

Formatted: Font: 10 pt

On multivariable analysis, women 20 years of age or younger were 2.5 folds more likely to test positive than those aged over 20 years; aOR 2.52, CI (1.31-4.90); p value 0.006) (Table 2 footnote).

For peer review only

For peer review only - http://bmjopen.bmj.com/site/about/guidelines.xhtml

DISCUSSION

This study highlights the high prevalence of Hepatitis B virus infection (11.8%) among pregnant women attending ante natal care in two hospitals in~~ the~~ post-conflict ~~region of~~ northern Uganda. Although the prevalence of both HBV and HIV infections in this region exceeds those in most other regions of Uganda that have not experienced prolonged civil conflict and internment in camps, no causal relationship between HBV infection and civil conflict can be inferred from this findings from a cross-sectional study. We also found that about 15% of the HBsAg positive mothers were also HBeAg positive. The prevalence of HBV infection was higher among women aged 20 years or younger (20%) ~~than among~~compared to the older women (8.7%). HIV infection among the study population was also high (9.3%). However there was no significant association between HIV infection and HBV infection among the pregnant women included in this study. ~~Thus about one in every eight pregnant women in Gulu is Hepatitis B infected.~~

The prevalence of HBV infection among pregnant women in this study is consistent with findings from a study in Nigeria ~~which found~~of a prevalence of 11%. The prevalence of HBeAg (33%) was however~~, and a~~ higher ~~HBeAg positivity of 33%~~in the Nigerian study.[12] The majority of people who get HBV infection after neonatal period tend to clear the virus over time. The natural history of hepatitis B infection follows three phases; immune tolerant, immune active and immune inactive phases. During the immune active phase when the virus is actively replicating and HBV DNA is high, HBeAg becomes positive and the individual is at higher risk of transmitting the virus. In the immune inactive phase, the individual has cleared the virus and HBsAg from blood and becomes less or not infectious to others unless they revert to the immune active phase. The liver enzymes particularly ALT are normal during the immune tolerant and

For peer review only

immune inactive phases. In our study the liver enzymes were largely within normal ranges and did not vary significantly between HBsAg positive and negative pregnant mothers. This may mean that most of our mothers were in the immune intolerant or immune inactive phases of their infections. In the Nigerian study where prevalence of HBeAg was up to 33%. It is probable that the mothers were in the immune active phases and could have had recent infections or were reverting from immune inactive to active phases[19]. ; it is therefore, conceivable for pregnant women to be at HBsAg positivity similar to the general population from which they come. Our findings add evidence to the fact that HBV infection rate might be high in the general population of northern Uganda. The finding in this study that 3 in 20 pregnant women with positive HBsAg are also HBeAg positive means that many the unborn babies in northern Uganda of these pregnant women are at high even higher risk of infection with HBV. The infants of all these HBsAg positive mothers will need immediate vaccination with HBV vaccine upon delivery. This is, however, not the practice in Uganda and that means the risk of infection is not adequately minimized in these infants. Children who contract HBV infections from their mothers are more likely to develop chronic HBV infection and progress to liver complications associated with active HBV infection including cirrhosis and hepatocellular carcinoma.

To demonstrate a need for a specific affirmative program to reduce the incidence of complications from chronic HBV infections in this community, we discuss our findings in context of HBV infections in Uganda as a whole. Review of the sentinel surveillance data shows that the HBV infections prevalence in this study is higher than HBV infection prevalence among the high risk HIV positive pregnant women (4.9%) in central Uganda[2017] and among HIV infected adult population (5%) in Rakai, south western Uganda[2118]. The prevalence of HBV infection of 18 - 24% in the general population in northern Uganda is in fact higher than in most

23

Formatted: Superscript

Formatted: Font: Bold, English (U.K.)

Formatted: Check spelling and grammar

Field Code Changed

Field Code Changed

Formatted: Check spelling and grammar

For peer review only

parts of Uganda, and higher amongst males than females,[14 22] and so the findings in this study for the pregnant population just mirrors the background female population prevalence in northern Uganda. It is important that special attention is paid to the high prevalence incidence of infection in this region in order to reduce the cost of care of chronic liver diseases including hepatocellular carcinoma in the future. We suggest that government of Uganda and development partners involved in healthcare planning and provisions in Uganda urgently introduce routine screening for HBV infection during pregnancy and vaccination at birth for the exposed infants, in the northern region in order to reduce incidences of peri-natal infections with HBV.

In this study, the prevalence of HBV infection was higher among the younger compared to the older women. This is in variance to findings from a study in Mauritania where there was no significant difference in the mean age of pregnant women who were HBsAg positive compared to those who were negative.[2320] Our finding is however, similar to results from the Uganda national sero-behavioral survey in 2005 which showed a prevalence of 8.8% in the age group 15-19 years of age and increments with age[2219] and in Rakai where positive HBsAg tests reached the highest at 8% among the age group 20-29 years of age.[2118] The high prevalence of HBV infection among the younger age group in this study and in the general Ugandan population may be related to the relatively high vulnerability of the younger women to sexually transmitted infections.[2421] In northern Uganda where people lived in the camps for more than 20 years, it is possible that these young women themselves acquired peri-natal HBV infections from their mothers who could have been exposed to sexually transmitted HBV during life in camps. A study by Råssjö et al, showed that females were more likely to be infected bydisposed to sexually transmitted infections (STIs) despite risky behavior being more common among males,[1722] and that Bbiological and social factors especially including unemployment and little

Formatted: Superscript
Field Code Changed
Formatted: Check spelling and grammar
Field Code Changed
Field Code Changed
Formatted: Check spelling and grammar
Formatted: Check spelling and grammar
Field Code Changed
Field Code Changed
Formatted: Check spelling and grammar
Field Code Changed
Formatted: Font: 12 pt, Check spelling and grammar

For peer review only - http://bmjopen.bmj.com/site/about/guidelines.xhtml

formal education contribute significantly to higher prevalence of STIs, including Hepatitis B, among adolescent girls. However, in thisour study there were no significant differences in employment status, education levels, marital status and number of sexual partners in the previous two years among HBsAg positive participants and those who were negative.

The risk of vertical transmission of HBV infection to the unborn child may be related to the effect of HBV infection on the mother; how she responds to the infection, the timing of the infection with respect to the current pregnancy and the immune status of the mother as well as the levels of HBV DNA.[2523] The probability of vertical infection is however, much increased when the mother is also positive for HBeAg.[3] In one study, vVertical transmission was seen in 65% of babies born to mothers who were positive for HBeAg and incompared to 9.1% for babies born to mothers who were negative for HBeAg.[2624] In Senegal, out of 21 infants born to HBsAg positive mothers 11 were HBsAg positive at birth;, and at 6 -7 months, five of these were still strongly HBsAg positive and developed antibodies to HBsAg, HBcAg or HBeAg.[2725] At present, pregnant women in Uganda are not routinely screened for HBsAg, and the exposed newborns are not immunized at birth against HBV infection. This high prevalence rate of HBsAg positivity among asymptomatic pregnant women in our study shows that there are many infants born who are at high risk of becoming chronic Hepatitis B carriers and dying of chronic liver disease at young adult age in future.

This study is not without limitation. We did not test for anti-HBc and HBV DNA; and so there could have been HBsAg negative individuals with isolated anti-HBc and occult HBV infection. However, a recent study among HIV infected pregnant women showed that pregnant women with isolated anti-HBc and occult HBV infection have very low HBV DNA levels and are thus at very low risk to transmit HBV to their infants.[2826] We also did not perform high resolution

Field Code Changed

Formatted: Check spelling and grammar

Field Code Changed

Field Code Changed

Formatted: Check spelling and grammar

Field Code Changed

Formatted: Check spelling and grammar

Field Code Changed

Formatted: Check spelling and grammar

For peer review only - http://bmjopen.bmj.com/site/about/guidelines.xhtml

abdominal ultrasound scans nor did we carry out serial liver enzyme tests to determine which mothers had active hepatitis B infections and may require treatment themselves. However, we referred every mother who tested positive for HBsAg to a competent physician for consultation.

IMPLICATIONS AND RECOMMENDATIONS

Formatted: Font: 12 pt, Bold

Formatted: Font: Bold

Government and development partners in health need to pay special attention to the high prevalence of infection in this region in order to reduce the cost of care of chronic liver diseases including hepatocellular carcinoma in the future. There is need to urgently introduce routine screening for HBV infection during pregnancy and provide vaccination at birth for the exposed infants in order to reduce incidences of peri-natal infections with HBV.

We recommend further studies to better characterize the pattern of HBV infections among the younger age groups 18 – 25 years. Results of such studies might provide guidance on appropriate methods of interventions to reduce incidence and prevalence of HBV among these younger populations.

CONCLUSIONS

There is a high prevalence of Hepatitis B infection among pregnant women attending antenatal care in Gulu and Lacor Hospitals. A high proportion of the HBsAg positive mothers are also HBeAg positive and may be at an increased risk of transmitting HBV infection to their unborn babies. These babies are at high risk of becoming chronic carriers of HBV infections and subsequently increasing the population pool of the virus.

~~pregnant women age ≤ 20 years are almost three times more likely to have Hepatitis B infection compared to the older women. There is need for screening of adolescent girls for Hepatitis B,~~

For peer review only - http://bmjopen.bmj.com/site/about/guidelines.xhtml

~~and an urgent program on immunizing babies born to mothers who are HBsAg positive in order to reduce the risk of vertical transmission to their unborn babies, reduce the future population risks of developing liver complications related to HBV infections including hepatocellular carcinoma.~~

Conflict of interest

We have read and understood BMJ policy on declaration of interests and declare that we have no conflict of interest regarding the publication of this article.

Authors' contributions

PB, EO and ADM participated in study design and drafting manuscript. CO participated in data collection. PB and EO analyzed the data. ADM edited and reviewed the final version of manuscript for important intellectual content and consistency. All authors read and approved the final manuscript.

Acknowledgements

The authors are grateful to the participants for their participation and cooperation ~~and without whom there would have been no data for this study~~. The authors are indebted to the laboratory staff particularly Ms Peace Amito who prepared the standard operating procedures for laboratory tests. The authors are also thankful to the research assistants who diligently counseled participants and collected data. This work would have been incomplete without the cooperation of the administrators of the two study hospitals to whom the authors are grateful.

Funding

For peer review only

This work was supported by **Training Health Researchers into Vocational Excellence in East Africa (THRiVE)**, grant number 087540 funded by the Wellcome Trust. Its contents are solely the responsibility of the authors and do not necessarily represent the official views of the supporting offices.

Exclusive Licence

"The Corresponding Author has the right to grant on behalf of all authors and does grant on behalf of all authors, a worldwide licence to the Publishers and its licensees in perpetuity, in all forms, formats and media (whether known now or created in the future), to i) publish, reproduce, distribute, display and store the Contribution, ii) translate the Contribution into other languages, create adaptations, reprints, include within collections and create summaries, extracts and/or, abstracts of the Contribution, iii) create any other derivative work(s) based on the Contribution, iv) to exploit all subsidiary rights in the Contribution, v) the inclusion of electronic links from the Contribution to third party material where-ever it may be located; and, vi) licence any third party to do any or all of the above".

For peer review only

For peer review only - http://bmjopen.bmj.com/site/about/guidelines.xhtml

References

1. Ching-Lung Lai, Man-Fung Yuen. Chronic Hepatitis B — New Goals, New Treatment. *N. Engl. J. Med.*, 2008;359(23):2488-2491.

Formatted: Font: Italic

2. Ott JJ, Stevens GA, Wiersma ST. The risk of perinatal Hepatitis B virus transmission: hepatitis B e antigen (HBeAg) prevalence estimates for all world regions. *BMC Infectious Diseases* 2012;12(131).

Formatted: Font: Italic

3. Stevens CE, Toy PT, Tong MJ, Taylor PE, Vyas GN, Nair PV, et al. Hepatitis B Virus transmission in the United States, Prevention by Passive-Active Ummunization. *JAMA* 1985;253(12).

Formatted: Font: Italic

4. Tassopoulos NC, Papaevangelou GJ, Sjorgen MH, Roumeliotou-Karayanis A, Gerin JL p, RH. Natural History of acute Hepatitis B surface antigen positive hepatitis in Greek adults. *Gastroenterology* 1987;92(June).

Formatted: Font: Italic

5. Beasley PR. Hepatitis B virus as the etiologic agent in hepatocellular carcinoma, epidemiologic considerations. *Hepatology* 1982;Suppl:21S-26S.

Formatted: Font: Italic

6. Alter MJ. Epidemiology of Hepatitis B in Europe and worldwide. *J Hepatol* 2003;Suppl(1):S64-9.

Formatted: Font: Italic

7. WHO. Introduction of hepatitis B vaccine into childhood immunization services: Management guidelines, including information for health workers and parents. *Department of Vaccines and Biologicals*. Geneva: World Health Organisation, 2001.

Formatted: Font: Italic

8. US Preventive Services Task Force. Screening for Hepatitis B Virus Infection in Pregnancy: U.S Preventive Task Force Reaffirmation Recommendation Statement. *Ann Intern Med* 2009;150:869-73.

Formatted: Font: Italic

9. Ministry of Health Republic of Uganda. Annual Health Sector Performance Report 2007-2008,. In: Health, editor: MoH, 2008.

10. Lavanchy D. Hepatitis B virus epidemiology, disease burden, treatment, and current and emerging prevention and control measures. *Journal of Viral hepatitis* 2004;11:97-107.

Formatted: Font: Italic

11. Andre FE, Zuckerman AJ. Protective efficacy of Hepatitis B vaccines in neonates. *Journal of Medical Virology* 1994;44:144-141.

Formatted: Font: Italic

12. Mbaawuaga E. M, Enenebeaku M.N.O, Okopi J. A, J.G Damen. Hepatitis B Virus (HBV) Infection among Pregnant Women in Makurdi, Nigeria. *African Journal of Biomedical Research* 2008;11(2008):155-159.

Formatted: Font: Italic

For peer review only - http://bmjopen.bmj.com/site/about/guidelines.xhtml

13. Rujumba J, Kwiringira J. Interface of culture, insecurity and HIV and AIDS: Lessons from displaced communities in Pader District, Northern Uganda. *Confl Health* 2010;4:18.

14. Ministry of Health Uganda. Uganda HIV/AIDS Sero-behavioural Survey 2004-2005. Calverton Maryland, USA: Uganda Ministry of Health and ORC Macro, 2006:125-132.

15. Ochola E, Ocama P, Orach CG, Nankinga ZK, Kalyango JN, McFarland W, et al. High burden of Hepatitis B infection in Northern Uganda: results of a population-based survey. *BMC Public Health* 2013;13(727).

16. St. Mary's Hospital Lacor. *Lacor Hospital Annual Report Financial Year 2012/2013*. Gulu: Lacor Hospital, 2013.

17. Råssjö EB, Mirembe F ED. Self reported sexual behaviour among adolescent girls in Uganda: reliability of data debated. *African Health Sciences* 2011;11(3):383-389.

18. WHO. *Preventing Mother-to-Child Transmission of hepatitis B : Operational field guidelines for delivery of the birth dose of hepatitis B vaccine*. Manilla: WHO, 2006.

19. Sorrel MF, Belongia EA, Costa J, Gareen IF, Grem JL, Inadomi JM, et al. National Institutes of Health Consensus Development Conference Statement: Management of Hepatitis B. *Ann Intern Med* 2009;150(2):104-10.

20. Maria F. Pirillo, Luciana Bassani, Elena A.P. Germinario, Maria Grazia Mancini, Joseph Vyankandondera, Pius Okong, et al. Seroprevalence of Hepatitis B and C Viruses Among HIV-Infected Pregnant Women in Uganda and Rwanda. *Journal of Medical Virology* 2007;79(2007):1797–1801.

21. Lara Stabinkski, Steven J Reynolds, Ponsiano Ocama, Oliver Laeyendecker, David Serwadda, Ron H G, et al. Hepatitis B virus and sexual behaviour in Rakai, Uganda. *J Med Virol* 2012;83(5):796-800.

22. Bwogi J, Braka F, Makumbi I, Mishra V, Bakamutumaho B, Nanyunja M, et al. Hepatitis B infection is highly endemic in Uganda: findings from a national serosurvey. *Afr Health Sci* 2009;9(2):98–108.

23. Mansour W, Malick F-ZF, Sidiya A, Ishaga E. Prevalence, Risk factors, and Molecular Epidemiology of Hepatitis B and Hepatitis Delta Virus in pregnant women and in patients in Mauritania. *Journal of Medical Virology* 2012;84(April):1186-1198.

24. Ministry of Health Uganda. Uganda AIDS Indicator Survey 2011: MoH, 2012.

25. Giles ML, Visvanathan K, Lewin SR, Sasadeuz J. Chronic Hepatitis B Infection and Pregnancy. *Obstetrical and Gynaecological Survey* 2012;67(1):37-44.

Formatted: Font: Italic

Formatted: Font: Italic

Formatted: Font: Italic

Formatted: Font: Italic

Formatted: Font: Italic

Formatted: Font: Italic

Formatted: Font: Italian (Italy)

Formatted: Font: Italic

Formatted: Font: Italic

Formatted: Font: Italic

Formatted: Font: Italic

Formatted: Font: Italic

For peer review only

26. Dwivedi M, Misra SP, Mishra V, Pandey A, Pant S, Singh R, et al. Seroprevalence of Hepatitis B infection during pregnancy and risk of perinatal transmission. *Indian journal of gastroenterology* 2011;30(2):66-71.

27. Phillippe R, Alassane D, Chiekh B, Souleymane M, D F. Perinatal Transmission of Hepatitis B virus in Senegal, West Africa. *Journal of Viral Immunology* 1993;6(1):65-73.

28. Khamduang W, Ngo-giang-huong N, Gaudy-graffin C, Jourdain G, Suwankornsakul W, Jarupanich T. Prevalence, Risk Factors, and Impact of Isolated Antibody to Hepatitis B core antigen and Occult Hepatitis B virus infection in HIV-1-Infected Pregnant Women. *Clinical Infectious Diseases* 2013:1-9.

1. Ching-Lung Lai, Man-Fung Yuen. Chronic Hepatitis B — New Goals, New Treatment. *N. Engl. J. Med.,* 2008;359(23):2488-91.

2. Ott JJ, Stevens GA, Wiersma ST. The risk of perinatal Hepatitis B virus transmission: hepatitis B e antigen (HBeAg) prevalence estimates for all world regions. *BMC Infectious Diseases* 2012;12(131).

3. Stevens CE, Toy PT, Tong MJ, Taylor PE, Vyas GN, Nair PV, et al. Hepatitis B Virus transmission in the United States, Prevention by Passive-Active Ummunization. *JAMA* 1985;253(12).

4. Tassopoulos NC, Papaevangelou GJ, Sjorgen MH, Roumeliotou-Karayanis A, Gerin JL p, RH. Natural History of acute Hepatitis B surface antigen positive hepatitis in Greek adults. *Gastroenterology* 1987;92(June).

5. Beasley PR. Hepatitis B virus as the etiologic agent in hepatocellular carcinoma, epidemiologic considerations. *Hepatology* 1982;Suppl:21S-26S.

6. Alter MJ. Epidemiology of Hepatitis B in Europe and worldwide. *J Hepatol* 2003;Suppl(1):S64-9.

7. WHO. Introduction of hepatitis B vaccine into childhood immunization services: Management guidelines, including information for health workers and parents. *Department of Vaccines and Biologicals*. Geneva: World Health Organisation, 2001.

8. US Preventive Services Task Force. Screening for Hepatitis B Virus Infection in Pregnancy: U.S Preventive Task Force Reaffirmation Recommendation Statement. *Ann Intern Med* 2009;150:869-73.

9. Ministry of Health Republic of Uganda. Annual Health Sector Performance Report 2007-2008,. In: Health, editor: MoH, 2008.

Formatted: Font: Italic

Formatted: Font: Italic

Formatted: Font: Italic

Formatted: Indent: Left: 0", First line: 0"

For peer review only - http://bmjopen.bmj.com/site/about/guidelines.xhtml

10. Lavanchy D. Hepatitis B virus epidemiology, disease burden, treatment, and current and emerging prevention and control measures. *Journal of Viral hepatitis* 2004;11:97-107.

11. Andre FE, Zuckerman AJ. Protective efficacy of Hepatitis B vaccines in neonates. *Journal of Medical Virology* 1994;44:144-41.

12. Mbaawuaga E. M, Enenebeaku M.N.O, Okopi J. A, J.G Damen. Hepatitis B Virus (HBV) Infection among Pregnant Women in Makurdi, Nigeria. *African Journal of Biomedical Research* 2008;11(2008):155-59.

13. Rujumba J, Kwiringira J. Interface of culture, insecurity and HIV and AIDS: Lessons from displaced communities in Pader District, Northern Uganda. *Conflict and health* 2010;4:18.

14. Ministry of Health Uganda. Uganda HIV/AIDS Sero-behavioural Survey 2004-2005. Calverton Maryland, USA: Uganda Ministry of Health and ORC Macro, 2006:125-32.

15. Ochola E, Ocama P, Orach CG, Nankinga ZK, Kalyango JN, McFarland W, et al. High burden of Hepatitis B infection in Northern Uganda: results of a population-based survey. *BMC Public Health* 2013;13(727).

16. St. Mary's Hospital Lacor. *Lacor Hospital Annual Report Financial Year 2012/2013*. Gulu: Lacor Hospital, 2013.

17. Maria F. Pirillo, Luciana Bassani, Elena A.P. Germinario, Maria Grazia Mancini, Joseph Vyankandondera, Pius Okong, et al. Seroprevalence of Hepatitis B and C Viruses Among HIV-Infected Pregnant Women in Uganda and Rwanda. *Journal of Medical Virology* 2007;79(2007):1797–801.

18. Lara Stabinkski, Steven J Reynolds, Ponsiano Ocama, Oliver Laeyendecker, David Serwadda, Ron H G, et al. Hepatitis B virus and sexual behaviour in Rakai, Uganda. *J Med Virol* 2012;83(5):796-800.

19. Bwogi J, Braka F, Makumbi I, Mishra V, Bakamutumaho B, Nanyunja M, et al. Hepatitis B infection is highly endemic in Uganda: findings from a national serosurvey. *Afr Health Sci* 2009;9(2):98–108.

20. Mansour W, Malick F-ZF, Sidiya A, Ishaga E. Prevalence, Risk factors, and Molecular Epidemiology of Hepatitis B and Hepatitis Delta Virus in pregnant women and in patients in Mauritania. *Journal of Medical Virology* 2012;84(April):1186-98.

21. Ministry of Health Uganda. Uganda AIDS Indicator Survey 2011: MoH, 2012.

22. Råssjö EB, Mirembe F ED. Self reported sexual behaviour among adolescent girls in Uganda: reliability of data debated. *African Health Sciences* 2011;11(3):383-89.

Formatted: Font: 12 pt, Italian (Italy)

Formatted: Italian (Italy)

Formatted: Font: 12 pt, Italian (Italy)

For peer review only - http://bmjopen.bmj.com/site/about/guidelines.xhtml

23. Giles ML, Visvanathan K, Lewin SR, Sasadeuz J. Chronic Hepatitis B Infection and Pregnancy. *Obstetrical and Gynaecological Survey* 2012;67(1):37-44.

24. Dwivedi M, Misra SP, Mishra V, Pandey A, Pant S, Singh R, et al. Seroprevalence of Hepatitis B infection during pregnancy and risk of perinatal transmission. *Indian journal of gastroenterology* 2011;30(2):66-71.

25. Phillippe R, Alassane D, Chiekh B, Souleymane M, D F. Perinatal Transmission of Hepatitis B virus in Senegal, West Africa. *Journal of Viral Immunology* 1993;6(1):65-73.

26. Khamduang W, Ngo-giang-huong N, Gaudy-graffin C, Jourdain G, Suwankornsakul W, Jarupanich T. Prevalence, Risk Factors, and Impact of Isolated Antibody to Hepatitis B core antigen and Occult Hepatitis B virus infection in HIV-1-Infected Pregnant Women. *Clinical Infectious Diseases* 2013:1-9.

Formatted: Space After: Auto

Formatted: Indent: Left: 0", Hanging: 0.5", Space After: Auto

For peer review only

STROBE 2007 (v4) Statement—Checklist of items that should be included in reports of *cross-sectional studies*

Section/Topic	Item #	Recommendation	Reported on page #
**Title and abstract**	1	(*a*) Indicate the study's design with a commonly used term in the title or the abstract	1
		(*b*) Provide in the abstract an informative and balanced summary of what was done and what was found	2
**Introduction**			**4**
Background/rationale	2	Explain the scientific background and rationale for the investigation being reported	4
Objectives	3	State specific objectives, including any prespecified hypotheses	5
**Methods**			**6**
Study design	4	Present key elements of study design early in the paper	6
Setting	5	Describe the setting, locations, and relevant dates, including periods of recruitment, exposure, follow-up, and data collection	6
Participants	6	(*a*) Give the eligibility criteria, and the sources and methods of selection of participants	6
Variables	7	Clearly define all outcomes, exposures, predictors, potential confounders, and effect modifiers. Give diagnostic criteria, if applicable	7
Data sources/ measurement	8*	For each variable of interest, give sources of data and details of methods of assessment (measurement). Describe comparability of assessment methods if there is more than one group	7
Bias	9	Describe any efforts to address potential sources of bias	
Study size	10	Explain how the study size was arrived at	6
Quantitative variables	11	Explain how quantitative variables were handled in the analyses. If applicable, describe which groupings were chosen and why	
Statistical methods	12	(*a*) Describe all statistical methods, including those used to control for confounding	9
		(*b*) Describe any methods used to examine subgroups and interactions	
		(*c*) Explain how missing data were addressed	
		(*d*) If applicable, describe analytical methods taking account of sampling strategy	
		(*e*) Describe any sensitivity analyses	
**Results**			10

For peer review only

Participants	13*	(a) Report numbers of individuals at each stage of study—eg numbers potentially eligible, examined for eligibility, confirmed eligible, included in the study, completing follow-up, and analysed	10
		(b) Give reasons for non-participation at each stage	
		(c) Consider use of a flow diagram	
Descriptive data	14*	(a) Give characteristics of study participants (eg demographic, clinical, social) and information on exposures and potential confounders	10
		(b) Indicate number of participants with missing data for each variable of interest	
Outcome data	15*	Report numbers of outcome events or summary measures	12
Main results	16	(*a*) Give unadjusted estimates and, if applicable, confounder-adjusted estimates and their precision (eg, 95% confidence interval). Make clear which confounders were adjusted for and why they were included	13
		(*b*) Report category boundaries when continuous variables were categorized	
		(*c*) If relevant, consider translating estimates of relative risk into absolute risk for a meaningful time period	
Other analyses	17	Report other analyses done—eg analyses of subgroups and interactions, and sensitivity analyses	14
**Discussion**			16
Key results	18	Summarise key results with reference to study objectives	16
Limitations	19	Discuss limitations of the study, taking into account sources of potential bias or imprecision. Discuss both direction and magnitude of any potential bias	19
Interpretation	20	Give a cautious overall interpretation of results considering objectives, limitations, multiplicity of analyses, results from similar studies, and other relevant evidence	17
Generalisability	21	Discuss the generalisability (external validity) of the study results	18
**Other information**			
Funding	22	Give the source of funding and the role of the funders for the present study and, if applicable, for the original study on which the present article is based	20

*Give information separately for cases and controls in case-control studies and, if applicable, for exposed and unexposed groups in cohort and cross-sectional studies.

Note: An Explanation and Elaboration article discusses each checklist item and gives methodological background and published examples of transparent reporting. The STROBE checklist is best used in conjunction with this article (freely available on the Web sites of PLoS Medicine at http://www.plosmedicine.org/, Annals of Internal Medicine at http://www.annals.org/, and Epidemiology at http://www.epidem.com/). Information on the STROBE Initiative is available at www.strobe-statement.org.

For peer review only
